# Health-Related Quality of Life in Metastatic Colorectal Cancer Patients Treated with Curative Resection and/or Local Ablative Therapy or Systemic Therapy in the Finnish RAXO-Study

**DOI:** 10.3390/cancers14071713

**Published:** 2022-03-28

**Authors:** Kaisa Lehtomäki, Hanna P. Stedt, Emerik Osterlund, Timo Muhonen, Leena-Maija Soveri, Päivi Halonen, Tapio K. Salminen, Juha Kononen, Raija Kallio, Annika Ålgars, Eetu Heervä, Annamarja Lamminmäki, Aki Uutela, Arno Nordin, Juho Lehto, Tiina Saarto, Harri Sintonen, Pirkko-Liisa Kellokumpu-Lehtinen, Raija Ristamäki, Bengt Glimelius, Helena Isoniemi, Pia Osterlund

**Affiliations:** 1 Faculty of Medicine and Health Technology, Tampere University, Arvo Ylpön Katu 34, 33520 Tampere, Finland; kaisa.lehtomaki@tuni.fi (K.L.); tapio.salminen@pshp.fi (T.K.S.); juho.lehto@tuni.fi (J.L.); pirkko-liisa.kellokumpu-lehtinen@tuni.fi (P.-L.K.-L.); 2 Department of Oncology, Tays Cancer Centre, Tampere University Hospital, Teiskontie 35, 33520 Tampere, Finland; 3 Department of Oncology, Kuopio University Hospital, Puijonlaaksontie 2, 70210 Kuopio, Finland; hanna.stedt@kuh.fi (H.P.S.); annamarja.lamminmaki@kuh.fi (A.L.); 4 Faculty of Health Sciences, University of Eastern Finland, Yliopistonranta 1A, 70210 Kuopio, Finland; 5 Department of Immunology, Genetics and Pathology, Uppsala University, Rudbecklaboratoriet, Dag Hammarskjölds Väg 20, 75185 Uppsala, Sweden; emerik.osterlund@igp.uu.se (E.O.); bengt.glimelius@igp.uu.se (B.G.); 6 Department of Oncology, South Carelia Central Hospital, Valto Käkelän Katu 1, 53130 Lappeenranta, Finland; timo.muhonen@mediexpert.fi; 7 Home Care, Joint Municipal Authority for Health Care and Social Services in Keski-Uusimaa, Sairaalakatu 1, 05850 Hyvinkää, Finland; leena-maija.soveri@keusote.fi; 8 Department of Oncology, University of Helsinki, Haartmaninkatu 8, 00290 Helsinki, Finland; paivi.halonen@hus.fi (P.H.); tiina.saarto@hus.fi (T.S.); 9 Department of Oncology, Helsinki University Hospital, Haartmaninkatu 4, 00290 Helsinki, Finland; 10 Department of Oncology, Central Finland Central Hospital, Keskussairaalantie 19, 40620 Jyväskylä, Finland; juha.kononen@docrates.com; 11 Docrates Cancer Centre, Docrates Hospital, Saukonpaadenranta 2, 00180 Helsinki, Finland; 12 Department of Oncology, Oulu University Hospital, Kajaanintie 50, 90220 Oulu, Finland; raija.kallio@ppshp.fi; 13 Department of Oncology, University of Oulu, Pentti Kaiteran Katu 1, 90570 Oulu, Finland; 14 Department of Oncology, Turku University Hospital, Hämeentie 11, 20520 Turku, Finland; annika.algars@tyks.fi (A.Å.); eeetu.heerva@tyks.fi (E.H.); raija.ristamaki@tyks.fi (R.R.); 15 Department of Oncology, University of Turku, Kiinanmyllynkatu 10, 20520 Turku, Finland; 16 Department of Transplantation and Liver Surgery, Abdominal Centre, Helsinki University Hospital, Haartmaninkatu 4, 00290 Helsinki, Finland; aki.uutela@hus.fi (A.U.); arno.nordin@hus.fi (A.N.); helena.isoniemi@hus.fi (H.I.); 17 Department of Surgery, University of Helsinki, Haartmaninkatu 8, 00290 Helsinki, Finland; 18 Palliative Care Centre, Tampere University Hospital, Teiskontie 35, 33520 Tampere, Finland; 19 Department of Palliative Care, Comprehensive Cancer Centre, Helsinki University Hospital, Haartmaninkatu 4, 00290 Helsinki, Finland; 20 Department of Public Health, University of Helsinki, Tukholmankatu 8B, 00290 Helsinki, Finland; harri.sintonen@helsinki.fi; 21 Department of Gastrointestinal Oncology, Tema Cancer, Karolinska Universitetssjukhuset, Eugeniavägen 3, 17176 Solna, Sweden; 22 Department of Oncology-Pathology, Karolinska Institutet, Solnavägen 1, 17177 Solna, Sweden

**Keywords:** metastatic colorectal cancer, metastasectomy, local ablative therapy, health-related quality of life, QLQ-C30, EQ-5D, 15D, QLQ-CR29, systemic therapy, chemotherapy

## Abstract

**Simple Summary:**

Metastatic colorectal cancer is the second most common cause of cancer death. Long-term survival and cure can be achieved after intensive treatments, including metastasectomy, i.e., the removal of all metastases. We wanted to clarify whether a patient health-related quality of life (HRQoL) was reduced by treatments that aimed to maximise metastasectomy rates, and whether HRQoL of treated patients is comparable to the general population. In a cross-sectional study of 444 patients (1751 questionnaires) in the RAXO-study population, we show that HRQoL of intensively treated patients, sometimes with multiple and multisite metastasectomies—usually combined with systemic therapy—remains at a high level during and after curative treatment and when compared with the general population. Good HRQoL was also seen during non-curative treatment from first- to later-lines, with an impaired HRQoL only at end-of-life. Thus, we should aim at maximising metastasectomies since they give long-term survival and sometimes cure with a high HRQoL.

**Abstract:**

Metastasectomy and/or local ablative therapy in metastatic colorectal cancer (mCRC) patients often provide long-term survival. Health-related quality of life (HRQoL) data in curatively treated mCRC are limited. In the RAXO-study that evaluated repeated resectability, a multi-cross-sectional HRQoL substudy with 15D, EQ-5D-3L, QLQ-C30, and QLQ-CR29 questionnaires was conducted. Mean values of patients in different treatment groups were compared with age- and gender-standardized general Finnish populations. The questionnaire completion rate was 444/477 patients (93%, 1751 questionnaires). Mean HRQoL was 0.89–0.91 with the 15D, 0.85–0.87 with the EQ-5D, 68–80 with the EQ-5D-VAS, and 68–79 for global health status during curative treatment phases, with improvements in the remission phase (disease-free >18 months). In the remission phase, mean EQ-5D and 15D scores were similar to the general population. HRQoL remained stable during first- to later-line treatments, when the aim was no longer cure, and declined notably when tumour-controlling therapy was no longer meaningful. The symptom burden affecting mCRC survivors’ well-being included insomnia, impotence, urinary frequency, and fatigue. Symptom burden was lower after treatment and slightly higher, though stable, through all phases of systemic therapy. HRQoL was high in curative treatment phases, further emphasizing the strategy of metastasectomy in mCRC when clinically meaningful.

## 1. Introduction

Colorectal cancer (CRC) is the third most common cancer worldwide with increasing incidence [1]. Approximately one-third of patients with metastatic CRC (mCRC) have oligometastatic disease, either synchronous or metachronous, and the treatment intention in these cases is often curative, with 5-year overall survival (OS) rates of 35–67% after metastasectomy (see review in Osterlund et al. [2]). The development of oncological treatments in mCRC has improved survival, reduced cancer-related symptoms and, above all, increased conversion to resectability [2,3], however, often at the expense of toxicity. Therefore, health-related quality of life (HRQoL) is an important outcome measure, along with OS and progression free survival (PFS), for both curative and non-curative strategies (with a goal of living longer and more comfortably, often designated as palliative chemotherapy) [4,5,6]. Although considered important, data on HRQoL in patients treated for cure are scarce. Most prospective studies have concentrated on patients included in non-curative systemic therapy trials [7], some on patients undergoing liver resection [8,9,10,11], and a few on cytoreductive surgery [12] or resection of multiorgan metastases [13].

HRQoL is a multidimensional concept including physical, functional, social, and emotional components. It is subjective and dynamic over time and, therefore, the individuals themselves should make the assessment regarding how these dimensions are affected by illness and treatment [14]. HRQoL can be measured with generic instruments, i.e., not disease-specific, such as the 15D [14] and the EuroQoL-5D-3L (EQ-5D) [15,16] and with cancer-specific instruments such as the EORTC QLQ-C30 [17] and the QLQ-CR29 [18,19]. Generic HRQoL measurements also allow comparison to a reference population, but reference population values are rarely present for cancer-specific questionnaires [20,21].

In the prospective RAXO study (NCT01531595, EudraCT 2011-003158-24), including 1086 Finnish treatable mCRC patients, the metastasectomy and/or local ablative therapy (LAT) rate was 37%, achieved after repeated centralized resectability assessment [2]. The median overall survival (mOS) was impressive if an R0/1-resection could be performed (80 months) and clearly shorter in patients who could not be resected (21 months in ‘systemic therapy only’ patients). An important outcome measure in the study was HRQoL, to clarify whether patients were harmed by the aggressive treatment plan that aimed to maximise resectability and survival. Secondary aims were to compare HRQoL at different timepoints after successful metastasectomy and/or LAT, during non-curative systemic therapy, and to compare these values to the Finnish general population.

## 2. Materials and Methods

### 2.1. Patients

**Figure 1:**
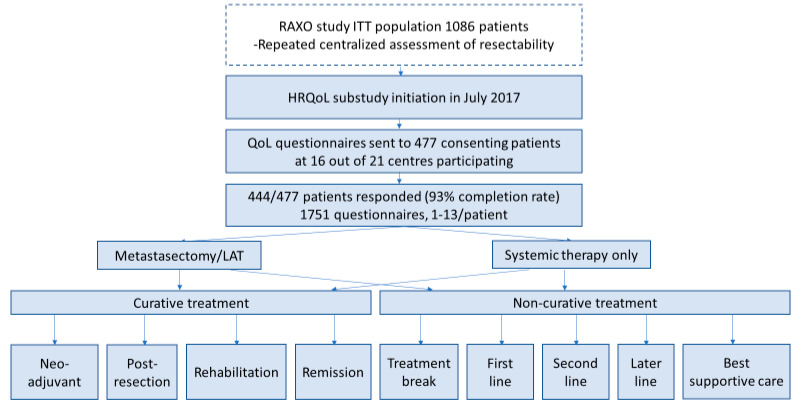
Study design, patient flow, Health-Related Quality of Life (HRQoL) questionnaires, and intervention with resectability assessments and metastasectomy and/or local ablative therapy (LAT) rates at a centralized multidisciplinary team (MDT) conference at a tertiary hospital in the Finnish nationwide RAXO-study. The exact patient flow between groups/phases and questionnaires per treatment phase are described in Table S1.

The nationwide Finnish RAXO study included 1086 mCRC patients between June 2012 and October 2018 [2]. The RAXO study was registered at ClinicalTrials.gov NCT01531621 and EudraCT 2011-003158-24. The HRQoL substudy was initiated after a protocol amendment in July 2017 (see supplement in [2]), when 477 patients out of 1086 were alive at 16 out of 21 participating centres, and consented to the HRQoL substudy (Figure 1). Of the consenting patients, 444 patients filled in at least one questionnaire and were thus eligible (questionnaire completion rate 93%), and in total 1751 questionnaires were collected. Questionnaires were given to patients at the hospital or sent out by mail multi-cross-sectionally (maximum 13 times). Patients were instructed to fill in the questionnaires just before a response evaluation and/or a doctor’s appointment. Time points were, thus, not treatment-phase-dependent or scheduled to baseline, at certain timepoints during a treatment phase, or after progression, as often is the case in longitudinal studies. This study was conducted in accordance with the Declaration of Helsinki and monitored independently. The protocol and amendment (supplement in [2]) were approved by the Ethics Committee at Helsinki University Hospital and all patients provided written informed consent, also for the current substudy.

### 2.2. Curative and Non-Curative Treatment Phases

The questionnaires were categorized into cross-sectional treatment phases. Treatment phases were defined as curative if the patient was (or became) resectable and metastasectomy/LAT was performed, and non-curative if systemic therapy was given with the goal of life-prolongation and palliation and no curative metastasectomy/LAT could be performed later (despite conversion aim in some patients).

Four curative treatment phases were identified: neoadjuvant (including more intense conversion therapy) before an R0/1-resection/LAT, post-resection/LAT (including adjuvant therapy) during the first 6 months after the R0/1-resection/LAT, rehabilitation (without treatment 6–18 months from an R0/1-resection/LAT or complete response [CR] to systemic therapy only), and remission (disease-free for more than 18 months from the last metastasectomy/LAT or CR). Patients completed the questionnaires in remission phase at a median of 38 months, maximum 94 months from the last metastasectomy/LAT.

The non-curative treatment phases were: first-line, second-line, and later-line (maximum 8 lines) systemic treatment, whether the intention was to allow subsequent metastasectomy/LAT or not but it was not reached, treatment breaks (patients who underwent an R2-resection/LAT, i.e., a macroscopically incomplete resection or second metastatic organ not resected, without any treatment for more than 2 months, or patients with a more than 2-month break from systemic therapy or a CR to systemic therapy within the first 6 months), and best supportive care (BSC after active oncological treatments were permanently stopped). A comprehensive characterization of patient flow for each group is provided in Figure 1, Table S1, and for patients answering multiple questionnaires, divided by metastasectomy/LAT or not in Figures S1 and S2. If a patient answered multiple times during one treatment phase, the mean values of all scores were used. The maximum number of completed questionnaires for an individual patient in the neo-adjuvant/conversion phase was 6, in the post-resection phase was 3, in the rehabilitation phase was 4, in remission was 5, in treatment break was 8, in first-line was 10, in second-line was 6, in later-line was 10, and in the BSC phase was 3.

### 2.3. Finnish Reference Population for 15D and EQ-5D-3L

Age- and sex-standardized samples of the Finnish population, which were obtained from the 2011 National Health Survey for the 15D (*n* = 4700) and from the Finnish Health 2000 Health Examination Survey for the EQ-5D-3L (*n* = 5217), were used as comparison groups [22,23].

### 2.4. HRQoL Instruments

For HRQoL evaluation, we used four different HRQoL questionnaires: the generic 15D [14] and EQ-5D-3L [24], which produce both index and profile data, and the disease-specific EORTC QLQ-C30 [17] and QLQ-CR29 [18] of which QLQ-C30 produces both index (global health status [GHS]) and profile measures, and QLQ-CR29 which produces only colorectal cancer-specific profile measures.

The 15D consists of 15 dimensions: moving, seeing, hearing, breathing, sleeping, eating, speech, excretion, usual activities, mental function, discomfort and symptoms, depression, distress, vitality, and sexual activity [14]. Each dimension has five levels from 1 (no problems) to 5 (extreme problems). For the 15D, we used the Finnish valuation algorithm for index scores (15D score) and dimension level values, both on a 0–1 scale [14]. The minimal clinically important difference (MID) of the 15D index score was ≥|0.015| in this study, which was considered slightly better or worse; a difference of >|0.035| was considered much better or worse [25]. Generally, a difference of ≥|0.20| is clinically important for 15D dimension level values and implies that there is a one level difference on the 5-level dimension scale.

The EQ-5D-3L health state index assesses health across five dimensions that include mobility, self-care, usual activities, pain or discomfort, and anxiety or depression. Each dimension has three possible levels (no problems, moderate problems, and extreme problems). The index score cannot be calculated if answers are missing. The maximum score of the EQ-5D is 1 [15]. The minimum score depends on the valuation algorithm used; the UK time-trade-off (TTO) tariff produces a minimum score of −0.594 [26]. The MID for this EQ-5D TTO score is ≥|0.08| [16]. The EQ-5D Visual Analogue Scale (VAS) provides an assessment of current health status on a vertical scale of 0–100, with 0 representing ‘worst imaginable health’ and 100 ‘best imaginable health’. The MID for the VAS mostly used is ≥|7| [16].

The EORTC QLQ-C30 (version 3.0) is a 30-item cancer-specific HRQoL questionnaire providing a GHS, five functioning scales (physical, role, social, emotional, and cognitive functions), three symptom scales (fatigue, nausea/vomiting, and pain) and six single-symptom items (dyspnoea, insomnia, appetite loss, constipation, diarrhoea, and financial difficulties) [17]. Each item is scored from 1 (not at all) to 4 (very much), except the global QOL scale which ranges from 1 (very poor) to 7 (excellent), and scores are linearly transformed to a 0–100 scale. The higher scores represent better functioning on functional scales and global QOL, whereas higher scores on the symptom scales signify more complaints. Items were calculated according to the EORTC scoring manual [27]. In the EORTC QLQ-C30, a |5–10| mean score difference is regarded as a small, but subjectively significant/clinically meaningful, a |10–20| point change is regarded as moderate, and a more than 20-point change as large [28]. The cut-off for MID used in this study was ≥|5|.

The EORTC QLQ-CR29 is a tumour-specific HRQoL questionnaire module for CRC patients, which is designed to complement the EORTC QLQ-C30 questionnaire [18]. It is psychometrically validated in CRC patients in different clinical situations that cover physical, psychosocial, and CRC-specific symptom-oriented questions. The questionnaire has sufficient validity and reliability to supplement the QLQ-C30 [19]. The QLQ-CR29 has five functional and 18 symptom scales. Scores are linearly transformed to provide a score from 0 to 100. Higher scores represent better functioning on the functional scales and a higher level of symptoms on the symptom scales.

Missing values in multiple-item questionnaires were replaced using multiple imputations, according to the manuals for the 15D, QLQ-C30, and QLQ-CR-29 questionnaires, but not for the EQ-5D.

### 2.5. Statistical Analysis

Results are presented as mean values with standard deviations and 95% confidence intervals (CI) for HRQoL scores in each treatment phase. Comparisons were performed with the non-parametric Mann–Whitney test for two-group-independent samples and the Wilcoxon signed-rank test for paired samples. MID was used with cut-offs as described above. We calculated proportions for the key demographic characteristics with Chi-square tests with Bonferroni correction per variable. We compared the 15D scores, EQ-5D scores, and the 15D profiles with those of the general population by means of Student’s independent samples t-test. The age and gender distributions of the population samples were weighted to correspond to those of the patients in each treatment phase. OS was estimated with Kaplan–Meier and compared with Cox regression, adjusted for age, sex, comorbidities, stage at CRC diagnosis, body mass index (BMI), and sidedness of the primary tumour. OS was calculated from the first questionnaire the patient completed (after inclusion to the HRQoL study) until death or last date of follow-up, with data cut-off of 30 April 2021. All *p*-values are two-sided, with values < 0.05 considered statistically significant.

## 3. Results

### 3.1. Study Population and Questionnaires

After re-consent, the first questionnaires were sent by mail to 477 patients alive at the participating centres (Figure 1).

The questionnaire completion rate was 93%, as 444 out of 477 patients answered the questionnaires at least once. Thirty-three patients were alive and declined inclusion at time of HRQoL substudy initiation (non-responders). There were some imbalances in, for example, Eastern Cooperative Oncology Group (ECOG) performance status in both metastasectomy/LAT and systemic therapy only subgroups compared with included patients (details of these imbalances are shown in Table S2). In total, 1751 questionnaires were answered between July 2017 and April 2021, with 188 patients (42%) answering once and 256 patients (58%) answering 2–13 times (Table S1). Baseline demographics for patients that underwent metastasectomy/LAT or received systemic therapy only are presented in Table 1, with more detailed information in Table S3.

Patients that were to have metastasectomy/LAT were slightly younger, had a better ECOG performance status, and more commonly had the primary tumour resected, a left-sided primary, one metastatic site, liver metastases, or a tumour that was *RAS*/*BRAF* wild-type, in comparison with systemic therapy only patients (Table 1). The primary tumour was never resected in 0.4% of the metastasectomy/LAT group and in 34% of the systemic therapy only group. Median time on active systemic treatment in the metastasectomy/LAT group was 8.9 months (range 0–59) and 16.4 months (0.2–53) in the systemic therapy only arm.

### 3.2. Multi-Cross-Sectional HRQoL Index Measures in Curative and Non-Curative Treatment Phases

**Figure 2:**
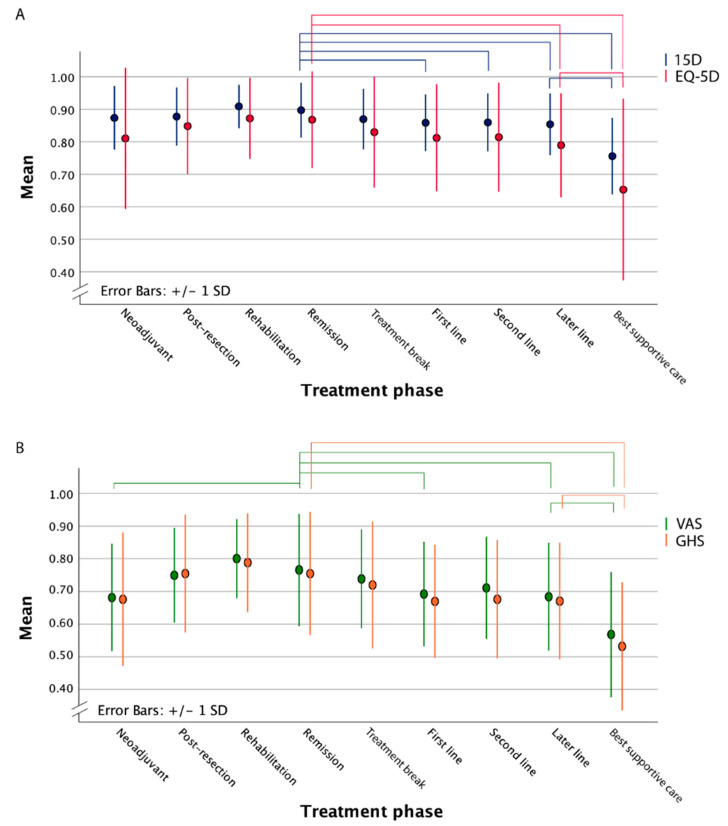
The mean health-related quality of life measured with the 15D and EQ-5D (**A**) and the VAS and QLQ-C30 global health (**B**) scores during treatment trajectory. Statistically significant differences between treatment phases are marked with brackets. Abbreviations: VAS = Visual Analogue Scale, GHS = Global Health Status.

The mean 15D scores were slightly higher throughout all treatment phases compared with EQ-5D TTO scores, and similarly, EQ-5D VAS scores were generally higher than QLQ-C30 GHS (Table 2, Figure 2).

HRQoL with any of the four instruments demonstrated only a few MIDs between the curative treatment phases, when the reference remission phase (disease-free more than 18 months from metastasectomy/LAT) was compared to neoadjuvant (including more intense conversion therapy), post-resection (0–6 months after metastasectomy/LAT including adjuvant-like therapy) or rehabilitation (6–18 months after metastasectomy/LAT) phases, respectively (Table 3, Figure 2). When the remission phase was compared to the neoadjuvant phase, MIDs were observed using the VAS and GHS (Table 3). When the neoadjuvant therapy group was divided according to treatment intent, i.e., given with conversion intent for a borderline/non-resectable tumour compared with neoadjuvant systemic therapy in an upfront resectable tumour, MIDs were noted for 15D and VAS (mean HRQoL difference in 15D of 0.033, EQ-5D 0.040, VAS 7.35, and GHS 2.39). When the remission or rehabilitation phases were compared with the post-resection phase, MIDs of 15D were noted.

Overall, 31 (13% of metastasectomy/LAT) patients had LAT, including percutaneous ablation or SBRT, in all but seven combined with a surgical metastasectomy during disease trajectory. Of the seven, three were disease-free for more than 18 months (two with percutaneous ablation of liver metastases and one with SBRT for lung metastases) and had HRQoL scores similar to those having major surgery.

The 15D, EQ-5D, VAS, and GHS showed MIDs when the remission phase was compared to the non-curative phases, including first-, second- and later-line systemic therapy (Table 3, Figure 2). When comparing ‘remission’ to ‘treatment break’, a MID was shown using the 15D.

When patients having first-line, non-curative systemic treatment were compared to those having second- or later-line treatment, MIDs for VAS were only observed between patients having first- and later-line therapy, however, without statistically significant differences. For the 15D, MID differed in a statistically significant way between patients having neoadjuvant treatment over those receiving first-line treatment. Minor changes between ‘treatment break’ compared to those in ‘first-line treatment’ were noted. When comparing patients having first-line treatment after metastasectomy/LAT, i.e., after non-re-resectable relapse with systemic therapy only patients, no MIDs were observed (mean 15D score difference of 0.022, EQ-5D 0.018, VAS 2.48, and GHS 4.12).

In the BSC phase, HRQoL was the poorest and differed from all other treatment phases, regardless of the instrument used (Table 2 and Table 3, Figure 2).

Compared with the general Finnish population standardized for age and sex, patient HRQoL values, measured as mean 15D and EQ-5D scores, stayed similar throughout all curative treatment phases (Table 4). Mean EQ-5D scores of CRC survivors were statistically significantly, but not clinically importantly, better in the rehabilitation and remission phases than those of the general population, and for the 15D, they were similar to those of the general population.

### 3.3. Longitudinal HRQoL Index Measures in Curative and Non-Curative Treatment Phases

When the same patients answered questionnaires in multiple curative treatment phases, i.e., longitudinally, no statistically significant differences were noted, with heat-maps for each patient presented horizontally in Figure S1.

If patients answered multiple questionnaires, both in conjunction with a curative treatment and during any non-curative treatment phase, i.e., after a non-re-resectable relapse, a statistically significant difference was noted for patients in remission compared to patients during second-line treatment (Figure S1). The HRQoL of patients with prior metastasectomy/LAT that answered multiple questionnaires only during non-curative phases was not statistically significantly different from that of patients answering both in curative and non-curative phases, presented in the lower section of Figure S1.

During the non-curative treatment phases, significant differences were noted for later-lines of treatment vs BSC, with heat-maps presented in Figure S2.

### 3.4. 15D Profile Measures

**Figure 3:**
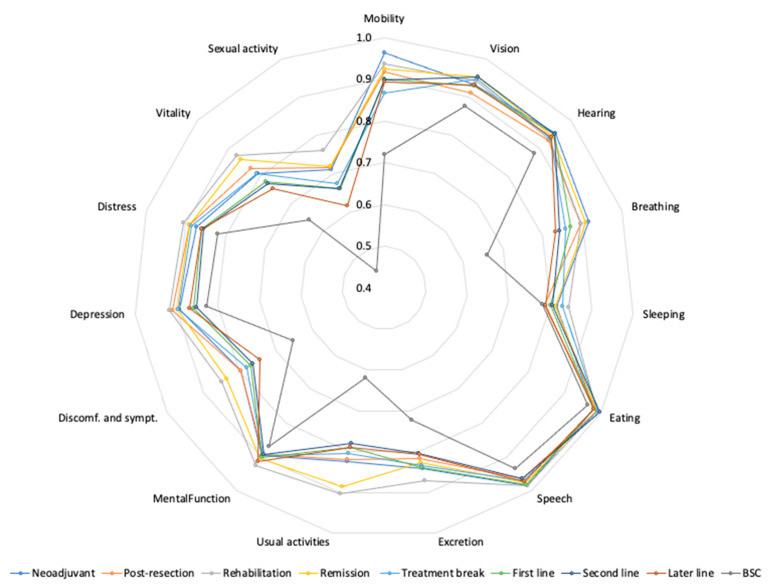
The mean 15D profile in different treatment phases. Abbreviations: Discomf. and sympt. = Discomfort and symptoms, BSC = Best Supportive Care.

The mean 15D profile showed that mobility, vision, hearing, eating, speech, and mental function sustained well during treatment trajectory, except for in the BSC phase, in which worse values were observed throughout (Figure 3). Breathing declined slightly during second- and later-line treatment. Sleeping was disturbed during all the treatment phases. Usual activities were disturbed before and after metastasectomy and during systemic treatment, but reverted during rehabilitation and remission. Discomfort and symptoms improved after curative treatment in the rehabilitation and remission phases. Sexual activity was most disturbed of all profiles and deteriorated with any systemic therapy and improved slightly in follow-up without treatment (Figure 3).

Compared with the age- and sex-standardized Finnish general population, the 15D demonstrated some significantly worse scores in several treatment phases (Table 4). In the neoadjuvant and post-resection phases, i.e., during active treatment with systemic therapy and metastasectomy/LAT, statistically significant impairments were noted for vision, excretion, usual activities, depression, distress, vitality, and sexual activity. The domains impaired after curative treatments in the rehabilitation and remission phases were sleeping, eating, excretion, depression, distress, vitality, and sexual activity.

Statistically significant impairment in comparison to the standardized Finnish population was noted during treatment break and first- to later-line treatment for mobility, vision, breathing, sleeping, eating, usual activities, depression, distress, vitality, and sexual activity (Table 4). Sexual activity was the dimension the most affected by CRC treatment. Mental functioning stayed stable throughout the treatment trajectory. Significantly worse profile scales were noted during the BSC phase.

### 3.5. QLQ-C30 and QLQ-CR29 Functioning Scales

Eleven functioning scales are reported and the higher the scores, the better the function. During curative treatment, cognitive functioning was sustained the best (Figure S3A). Physical, role, emotional, and social functioning were affected during the neoadjuvant treatment, but improved during post-resection, rehabilitation, and remission phases. Body image and weight stayed relatively stable during the curative treatment phases, whereas sexual interest was strongly disturbed in both sexes, more severely in women.

During non-curative treatment, there were no significant changes in the functioning scales between lines of systemic treatment (Figure S3B). Again, cognitive functioning was preserved the best, while physical, role, emotional, and social functioning were also maintained during active treatment and were slightly better during the treatment break phase. Patients receiving BSC reported a more pronounced decline in functioning scales than the patients in other treatment phases did.

Functioning scale summary (sum of QLQ-C30 physical, role, emotional, cognitive, and social functioning presented in Figure S3) shows the mean values of 413–420 during the neoadjuvant therapy/post-resection phases and 436–441 after the curative treatment. In the non-curative treatment phases, the functioning scale summary was 396–401 during first- to later-line treatment, 407 during the treatment break, and 302 during BSC. No statistically significant differences were noted between the curative treatment phase comparisons or the non-curative treatment phase comparisons, apart from BSC. When comparing the remission phase to any non-curative treatment phase, statistically significantly better scores were noted.

### 3.6. QLQ-C30 and QLQ-CR29 Symptom Scales

**Figure 4:**
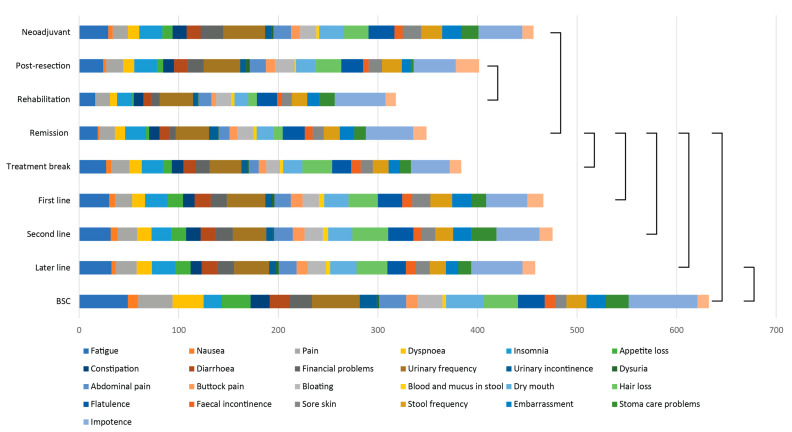
Symptom burden as sum of 26 symptom scales measured with the QLQ-C30 and QLQ-CR29 during different treatment phases. Statistically significant differences between treatment phases are marked with brackets. Abbreviations: BSC = Best Supportive Care.

Symptom burden (the sum of means for the 26 symptom scales, the lower the better) per treatment phase is shown in Figure 4. The symptom burden was higher during the neoadjuvant and post-resection phases compared with the rehabilitation and remission phases (Figure 4, Table 3). When the remission phase was compared to any non-curative treatment phase, statistically significantly lower scores were noted. The worst scores were again seen in the BSC group.

Symptoms that most affected patient well-being during the curative treatments were impotence, urinary frequency, and fatigue (Figure S4A). Impotence, urinary frequency, hair loss, and fatigue were the most reported symptoms affecting HRQoL during any systemic treatment (Figure S4B).

### 3.7. First 15D Index Score and QLQ-C30 Physical Functioning as Prognostic Markers

Forty-five percent of the first questionnaires were filled in during the first-line or neoadjuvant treatment phases, 29% after curative metastasectomy or LAT, and the rest during second-line, later-line, or treatment breaks, thus including guarantee-time bias.

Median OS from the first HRQoL measurement until death or censoring was 29 months (95% CI 23–35) in patients with a 15D score under the median (<0.90) and 36 months (95% CI 28–44) in those with scores above the median (≥0.90) (Figure S5A), with a hazard ratio (HR, adjusted for age, sex, comorbidities, stage at CRC diagnosis, BMI, and sidedness of primary) of 0.58 (95% CI 0.41–0.82). Similarly, mOS was 28 months (95% CI 24–32) in patients with physical functioning scale below the median (<80) and not reached if above the median (≥80) (Figure S5B), with an HR of 0.56 (95% CI 0.37–0.84).

### 3.8. Association of Remission Phase Index Scores with Demographic Factors, Metastatic Sites and Metastasectomies

Associations between 15D, EQ-5D, VAS, and GHS scores during the remission phase and demographic factors are presented in Table S4. Generally, worse scores were observed, irrespective of the questionnaire, for patients having comorbidities, higher BMI, and if radiotherapy was provided.

Associations between metastatic sites and metastasectomy/LAT showed worse GHS if liver metastases were present at baseline or developed during the disease trajectory, and for liver procedures (Table S5). Worse EQ-5D was associated with no performed lung resection/LAT, whereas 15D and VAS scores showed no significant associations.

## 4. Discussion

We show that the HRQoL of intensively-treated mCRC patients, sometimes including multiple and multisite metastasectomies, generally combined with systemic therapy, remains at a high level both during and after the curatively intended treatments. This high level is also observed after the treatments when compared with the Finnish general population, highlighted by higher index scores for EQ-5D and similar for 15D. Thus, in addition to the long OS observed in the RAXO study [2], this HRQoL substudy, demonstrating a high HRQoL, supports maximising metastasectomy/LAT in mCRC patients. HRQoL also remained stable and at a reasonably high level during first- to second- and later-line treatments, and when the aim was no longer curative, and declined only in the BSC phase, when tumour-controlling therapy was no longer meaningful.

Mean HRQoL index scores, from the neoadjuvant treatment phase to long-term remission, ranged from 0.89 to 0.91 with the 15D, from 0.85 to 0.87 with the EQ-5D, from 68 to 80 with the VAS, and from 68 to 79 with the GHS. Surprisingly, we noted similar scores with the 15D and even higher EQ-5D scores in the rehabilitation and remission phases than for the age- and sex-standardized general Finnish population. However, all results of comparisons of patient EQ-5D scores with those of the population should be interpreted with caution, as the scores were measured in 2000 for the general population and in 2017 or later in this study. A Finnish general population study reported that there was a significant improvement in the EQ-5D of older age groups in the population from 2000 to 2011 [23] and most of our patients belong to these older age groups. Of note is that the 15D scores in the general population are from 2011 [22] when this study started. Yet, it seems as if the HRQoL among CRC survivors is quite similar to that of the general population as was also found in previous studies [29,30].

The scores reported here are in line with those from several studies in patients with localised CRC after primary surgery only, not undergoing intense chemotherapy and more extensive surgery than removal of the primary tumour [31,32,33]. For example, in one cross-sectional study of 1294 CRC survivors [32], GHS was comparable to the present study, but in that study, more than half of the patients were treated with primary surgery alone, which is substantially less than systemic treatment in virtually all patients and mean 2.5 surgeries per patient in this study.

HRQoL after metastasectomy in mCRC has been reported, however, mostly for patients with single-site metastases [8,9,11,12]. In a study of 24 patients undergoing cytoreductive surgery with hyperthermic intraperitoneal chemotherapy for peritoneal metastases, HRQoL, measured with the SF-36 and GHS, was comparable to the general population at 2 years [12]. Liver resection transiently deteriorates HRQoL, with recovery within one year [8,9,11]. Long-term HRQoL after liver resection assessed with the GHS was slightly higher than in our study [10]. HRQoL measured using the EQ-5D score appeared to be better in patients undergoing re-resection than receiving systemic therapy after relapse [8]. We also noted maintained EQ-5D in re-resected patients, performed in one-third of our patients.

To our knowledge, there is only one published study reporting HRQoL data after multisite and multiple metastasectomies [13]. In the study (*n* = 26), HRQoL (measured with GHS, QLQ-LMC21, FACT-HEP) declined transiently after the interventions with recovery within 1 year post-resection, without impairment after sequential resections [13]. This study supports our results, showing preservation of HRQoL after multisite and multiple resections for mCRC, and of active evaluation of resectability of both hepatic and multisite extrahepatic metastases. We observed no differences in HRQoL using any of the four index scores during the remission phase according to type of metastatic site, number of metastatic sites, organ of metastasectomy/LAT (including whether an LAT was required or not, or in the two patients having a percutaneous LAT-procedure only), or after multiple procedures compared to a single procedure.

During perioperative systemic treatment aiming at cure, the HRQoL with GHS reported here is comparable to that reported by Hamidou et al. [34]. During the induction phase, clinically important differences in two out of four instruments (15D and VAS) were noted if the intent was conversion versus strictly neoadjuvant (only aiming to kill micrometastases to decrease recurrence risk), implying that more intense treatment and advanced tumour did not have any major impact on the index scores.

HRQoL scores during non-curative treatment phases were clinically importantly inferior to those in the remission phase, but were surprisingly high compared with the general population, comparisons rarely reported in literature [29]. In the present study, HRQoL was measured during all lines of therapy, with more than half of the patients answering longitudinally at least twice during non-curative therapy phases which, to the best of our knowledge, has not been reported before. Most intriguing was that the mean HRQoL scores stayed stable both in multi-cross-sectional and longitudinal analyses during first-, second-, and later-line therapy, with a drop as late as in the BSC phase. In the literature, first-line [35,36], first-line maintenance [37], second-line [36,38], and later-line [36,39] scores are in agreement with the present study. HRQoL measurements have often been collected in phase III trials during the past about 30 years but the information published in the reports has been limited and mostly restricted to a global measure, especially missing in second- and later-lines, where attention to HRQoL is particularly high [7]. A common conclusion of phase III trial results in mCRC is preserved global HRQoL despite more intense treatment, generally meaning more toxicity [40], in line with index scores reported in this study.

Metastasectomy is the gold standard in patients with upfront resectable metastases in mCRC [3]. It is frequently combined with perioperative chemotherapy since this reduces recurrence rates [41]. Local ablative treatment (LAT) is an acceptable alternative therapy, sometimes the only possible treatment, for patients who are poor candidates for surgery and for patients with post-metastasectomy recurrence [3,42,43,44,45,46], the main inclusion criteria for LAT only strategy in the RAXO study. It is also often combined with metastasectomy to obtain a radical procedure when surgery cannot leave a sufficient amount of normal liver behind [42], constituting the majority of patients with LAT in this study. In the non-curative setting, systemic therapy as the only strategy leads to median OS of 15–21 months in population-based patient series [2,47]. The CLOCC trial compared systemic therapy with or without radiofrequency ablation and demonstrated that adding a local treatment might prolong OS in patients with non-resectable CRC liver metastases [48]; contrary to the aim of cure with LAT in this study. Currently, there is no clear evidence supporting the superiority of one LAT technique over the other [43]. In oligometastatic mCRC, LAT of lung or liver metastases offers both disease-free and chemotherapy-free intervals [3] with a favourable safety profile [43,44,45,46], which may contribute to better QoL compared with systemic therapy only [49]. In our study, being the tradition among Finnish liver surgeons, LAT was mainly used in combination with metastasectomy to obtain macroscopic removal of all visible tumour if this was not possible with surgery only, and only seven out of 31 patients had LAT only. The study design does not enable any evaluation of HRQoL related to the metastasectomy or LAT separately in the remission phase, as only three patients answered the questionnaires in the LAT only group.

The mean values for GHS collected during treatment breaks lasting for more than 2 months were slightly higher than during active treatment and symptom burden was not statistically lower, with no difference in functioning scale sums. A chemo-holiday is, by most oncologists, considered to be of great value for improvement of well-being (i.e., HRQoL), a conclusion which is questioned by our findings. Another explanation is that the instruments are not sensitive enough to detect any differences that may be present.

As expected, more pronounced impairment in HRQoL was observed during the BSC phase, as observed previously [29,50], with findings in line with a recent review [51]. Nearly all patients in the BSC group have progressive disease, and many have a poor performance status preventing further oncologic therapies. Information on the quality of the palliative care received was not available.

In the present study, after multi-organ and multiple resections in one-third of patients, the functioning scales with physical, role, emotional, cognitive, and social functioning measures were slightly inferior in the remission phase compared with those reported after surgery for localised CRC disease [32], or after single liver resection [10], but follow-up time was significantly shorter in the present study. Generally, the scores of the functioning scales of the QLQ-C30 were better in the curative treatment phases than in the non-curative, with no significant impairment until the BSC phase.

As the 15D produces functionality profile data (as mobility, vision, hearing, breathing, sleeping, eating, and speech), we compared the profiles seen here in the different treatment phases to those reported previously on Finnish CRC and mCRC patients, and found them astonishingly similar [29], giving a verification of repeatability in multi-cross-sectional data collection.

Most CRC metastases that later can be resected do not cause any physical symptoms, and, therefore, surgery itself does not alleviate symptoms. Instead, metastasectomy saves patients from disease progression and, if the disease does not recur, from multiple lines of systemic treatment. The symptom burden is otherwise caused by the cancer itself and by the systemic treatments. In clinical routine it may sometimes be difficult to differentiate between these two, but it is impossible when you only have questionnaire data. In our study, the symptom burden, consisting of the sum of 26 symptoms scales, was somewhat higher during the non-curative treatment phases than during the curative treatment phases, suggesting that the cancer contributes to the burden and, naturally, this was worst during the BSC phase when the cancer was progressive. During the curative treatment phases, symptom burden was highest during the neoadjuvant therapy phase and diminished through post-resection to rehabilitation/remission phases. The symptom burden remained similar during systemic treatment, whether first-, second- or later-line, indirectly supporting the use of systemic treatment in several lines since these prolong survival [3,7]. A single index score may not capture small differences in symptom burden, particularly since some symptoms do not affect the patients’ general well-being. Of the index scores, 15D seems most in line with the symptom burden findings.

Hinz et al. showed among cancer survivors that the global QoL is not the sum of its parts. Specifically, they showed that while the index score GHS was nearly equal to that of the general population during active treatment, the mean scores of functioning scales, symptom scales, and symptom items showed markedly worse QoL [20]. This is in line with our findings with 15D index versus profiles in comparison to the general population showing the importance of reporting profile score comparisons on top of index scores.

Symptoms more profoundly affecting CRC survivors’ well-being, in this study as well as in others, are insomnia, impotence, urinary frequency, and fatigue [32,52]. Sexual activity, sexual interest, and dyspareunia were also strongly disturbed in both genders, but more severely in women. Compared to a standardized general population, sexual activity was impaired in all phases of curative and non-curative treatment and was the most affected dimension of the 15D. This is in line with literature showing that sexual impairment is a major adverse condition of cancer and its treatments [29,53,54]. Our results show poor anxiety scores, though with improvement after longer follow-up. This is supported by earlier findings from ‘fear of cancer recurrence’ studies. ‘Fear of cancer recurrence’ is a relevant aspect causing anxiety and decreased functioning in resected mCRC patients. After resection of the primary cancer, it decreases with time after diagnosis [55]. Most CRC survivors are at least ‘a little worried’ about a CRC recurrence [56].

During systemic treatment the most common complaints are fatigue and insomnia [34,35,37,38], as shown also in this study. Dry mouth, hair loss, and flatulence were also common during systemic treatment.

Sexual complaints, urinary frequency, insomnia, fatigue, and anxiety need to be discussed with the patients and symptom management should be addressed in all phases of treatment for mCRC. The need for survivorship programmes is recognized. Cognitive therapy is less studied but evidence supports psychoeducation, psychosocial support, and nurse-led supportive care [57], to relieve anxiety and depression, and exercise interventions for fatigue and cardiopulmonary fitness. Unfortunately, there is limited evidence for interventions to address sexual concerns [57].

It is well established that poorer HRQoL, when measured cross-sectionally [31,32,52], is a prognostic marker for shorter survival, a factor also observed in this study with the 15D score. This should be interpreted with caution as it includes guarantee-time bias due to the study setting. The strongest association has been shown between the QLQ-C30 physical functioning scale and mortality [32,58,59,60], similar to our results. The association of poorer HRQoL, physical functioning, and ECOG PS is unavoidable [59], and ECOG is probably one of the strongest prognostic factors in mCRC [61], making these associations probable. Lower HRQoL associates with more advanced disease stage, metastatic disease, other cancers, chemotherapy, radiotherapy, and current stoma [32]. These factors were also associated with impaired HRQoL in our data, thus making associations with survival understandable.

A major limitation of this study is the study design in which HRQoL assessment started mid-study and was measured cross-sectionally at multiple time-points, with nearly half of the patients answering only once. Cross-sectional data have limitations, with guarantee-time bias being one of the most challenging, accentuated in rehabilitation, remission, and later-line treatment phases. Missing data at later time points may be related to treatment-related toxicities, tumour progression, and difficulties in completing questionnaires. Some patients answered questionnaires more than once during the same treatment phase and were then given a mean score that could result in problems with interpretation of the scores. A patient could be captured in both the curative phase and, after a non-re-resectable relapse, also in the non-curative treatment phase. Another major limitation of the study is that we had very few baseline assessments before any treatment for mCRC and, thus, the potential prognostic value of HRQoL measured with any of the scales are difficult to interpret. A further limitation to consider is the non-optimal comparison to EQ-5D population scores, as discussed earlier. Based on these major limitations concerning the study design, our results cannot be generalized to all mCRC patients undergoing curative or non-curative therapy.

A strength of the study is the questionnaire completion rate of 93%, in comparison with 73–91% for CRC patients in other cross-sectional studies [30,31], and clearly higher than the 60–70% reported in longitudinal studies at later time points during first or later line treatment [34,38]. Another strength is that we captured HRQoL data throughout the disease trajectory from mCRC diagnosis through multiple curative phases and non-curative treatments to BSC. One factor supporting the reliability of our cross-sectional results during multiple treatment phases is the repeatability when compared to previously published studies assessing multiple treatment phases, especially Färkkilä’s work [29]. Additionally, as some patient questionnaires were recorded multiple times during one treatment phase with random timing, this illustrates the different phases more reliably than a longitudinal study with significant drop-out from the second assessment and onwards. The definition of treatment phases, rather than time points, illustrates the curative phases more reliably as patients facing multiple resections were recorded multiple times in the different phases; the HRQoL assessment points were always determined based on the last metastasectomy/LAT. Another strength is the use of four different HRQoL questionnaires producing both generic and disease-specific indexes and profile scores, enabling a broader assessment of HRQoL and easy comparison between studies. An inherent limitation of all HRQoL assessments is the validity of the instruments. We only used ‘well-validated questionnaires’, however, the lack of major differences in scores between groups of patients being treated very differently and with marked differences in survival outcomes always indicate that the questionnaires do not properly cover what we want them to reflect. Finally, this prospective study, having thorough data collection, is representative of the whole nation. In addition, we have a representative general reference population with age- and sex-matching for each treatment phase separately.

## 5. Conclusions

HRQoL is one of the most important outcome measures, along with OS, in cancer studies. In a multi-cross-sectional setting, with its caveats, we were able to show high average HRQoL after curative treatment. Cross-sectionally measured HRQoL was also good during non-curative treatment phases from first- to later-line treatments but was clearly lower in the BSC phase. In symptom scales, several factors, including sexual aspects, urinary frequency, fatigue, distress, and insomnia impair HRQoL, despite having no negative effect on the index scores. Efforts should be made to decrease symptom burden as much as possible. The present strategy, with a goal of metastasectomy in mCRC whenever clinically reasonable, can continue to be the recommended strategy as it improves long-term survival and may even be curative, while providing HRQoL similar to that seen in the general population. More prospective longitudinal data is, however, needed to validate our findings.

## Figures and Tables

**Table 1:** Patient demographics according to metastasectomy and/or locally ablative therapy (LAT) performed during disease trajectory or systemic therapy only are given.

		All Patients		Curative Metastasectomy/LAT		Systemic Therapy Only	
		*n* = 444		*n* = 247		*n* = 197	
Age	Median (range)	64.7	(24.3–87.8)	64.3	(25.0–81.5)	66.2	(24.3–87.8)
	≤70	322	73%	189	77% *	133	68%
	>70	122	28%	58	24%	64	33% *
Sex	Male	258	58%	142	58%	116	59%
	Female	186	42%	105	43%	81	41%
ECOG	PS 0	172	39%	115	47% *	57	29%
	PS 1	235	53%	120	49%	115	58% *
	PS 2–3	37	8%	12	5%	25	13% *
Charlson comorbidity index	0	360	81%	204	83%	156	79%
	1 to 2	82	19%	42	17%	40	20%
	3 to 5	2	1%	1	0%	1	1%
Smoking	No	284	86%	159	87%	125	83%
	Yes	48	15%	23	13%	25	17%
BMI	≥20	412	93%	227	92%	185	94%
	<20	32	7%	20	8%	12	6%
Presentation §	Synchronous	298	67%	155	63%	143	73% *
	Early metachronous	38	9%	21	9%	17	9%
	Late metachronous	108	24%	71	29% *	37	19%
Primary tumour	Right colon	109	25%	50	20%	59	30% *
	Left colon	181	41%	116	47% *	65	33%
	Rectum	153	35%	81	33%	72	37%
	Multiple	1	0%	0	0%	1	1%
Surgery primary tumour	Surgery upfront	314	71%	196	79% *	118	60%
	No or later surgery	130	29%	51	21%	79	40% *
Prior adjuvant therapy	No adjuvant	338	76%	177	72%	161	82% *
	Fluoropyrimidine	45	10%	27	11%	18	9%
	Oxaliplatin-containing	61	14%	43	17% *	18	9%
Radiotherapy for rectal cancer	No	380	86%	208	84%	172	87%
	Preop 5x5Gy	27	6%	22	9% *	5	3%
	Chemoradiation	30	7%	13	5%	17	9%
	Palliative	7	2%	4	2%	3	2%
Metastatic sites	1	284	64%	195	79% *	89	45%
	2	113	26%	43	17%	70	36% *
	3 to 6	47	11%	9	4%	38	19% *
	Liver	326	73%	201	81% *	125	64%
	Lung	118	27%	46	19%	72	37% *
	Peritoneal	55	13%	21	9%	34	19% *
	Distant lymph nodes	95	21%	17	7%	78	40% *
	Other	53	12%	25	10%	28	14%
Molecular status	*RAS*/*BRAF* wild-type	153	38%	94	43% *	59	32%
	*KRAS*/*NRAS* mutant	222	55%	120	54%	102	56%
	*BRAF* mutant of tested	29	10%	7	4%	22	15% *

* *p*-value < 0.05. § early metachronous = 2–12 months from diagnosis of metastatic disease, late metachronous = more than 12 months from diagnosis. Abbreviations: ECOG PS = Eastern Cooperative Oncology Group Performance Status, BMI = Body Mass Index, *KRAS* = Kirsten rat sarcoma viral oncogene homolog, *NRAS =* Neuroblastoma rat sarcoma viral oncogene homolog, *BRAF* = v-raf murine sarcoma viral oncogene homolog B1.

**Table 2:** Mean health-related quality of life scores during treatment trajectory.

Treatment Phase	Patients	15D			EQ-5D			VAS			GHS		
	*N*	Mean	SD	95% CI	Mean	SD	95% CI	Mean	SD	95% CI	Mean	SD	95% CI
**Curative**													
Neoadjuvant	56	0.889	0.096	0.864–0.915	0.834	0.204	0.776–0.893	67.8	16.2	63.1–72.5	68.3	20.2	62.5–74.1
Post-resection	58	0.876	0.090	0.853–0.900	0.848	0.148	0.808–0.888	75.0	14.5	71.0–78.9	75.6	17.9	70.8–80.5
Rehabilitation	61	0.909	0.069	0.891–0.927	0.872	0.125	0.839–0.905	80.0	12.1	76.8–83.3	78.8	15.1	74.8–82.8
Remission	132	0.895	0.092	0.879–0.911	0.874	0.139	0.849–0.898	77.9	15.0	75.2–80.5	75.5	18.8	72.2–78.8
**Non-curative**													
Treatment break	87	0.869	0.091	0.849–0.888	0.830	0.170	0.791–0.869	73.9	15.1	70.4–77.4	71.9	19.3	67.5–76.3
First line	169	0.860	0.090	0.850–0.880	0.810	0.160	0.780–0.840	69.2	16.0	66.4–72.0	66.8	17.3	63.8–69.9
Second line	115	0.857	0.088	0.841–0.874	0.812	0.168	0.779–0.845	71.1	15.6	68.0–74.2	67.6	18.1	64.1–71.2
Later line	106	0.853	0.095	0.834–0.871	0.790	0.159	0.759–0.820	68.4	16.5	65.2–71.6	67.1	17.8	63.6–70.5
BSC	35	0.763	0.123	0.720–0.805	0.653	0.279	0.555–0.750	56.8	19.2	49.9–63.8	54.2	20.7	47.0–61.4

Abbreviations: SD = Standard Deviation, CI = Confidence Interval, VAS = Visual Analogue Scale, GHS = Global Health Status, BSC = Best Supportive Care. For definition of the treatment phases, see materials and methods.

**Table 3:** Comparisons of index measures between different treatment phases.

	15D		EQ-5D		VAS		GHS		Symptom Burden		Functioning Scale Sum	
	∆	*p* Value	∆	*p* Value	∆	*p* Value	∆	*p* Value	∆	*p* Value	∆	*p* Value
**Curative vs. Curative**												
Remission vs. Neoadjuvant	0.005	0.683	0.039	0.537	**10.07**	**<0.001**	**7.14**	**0.019**	−86	**0.002**	23	0.071
Remission vs. Post-resection	**0.018**	0.115	0.025	0.263	2.9	0.106	−0.16	0.693	−92	0.052	16	0.331
Remission vs. Rehabilitation	−0.014	0.666	0.002	0.771	−2.17	0.670	−3.3	0.550	29	0.649	−5	0.486
Rehabilitation vs. Post-resection	**0.033**	0.063	0.024	0.367	5.07	0.06	3.15	0.373	−121	**0.029**	21	0.177
**Curative vs. Non-curative**												
Remission vs. Treatment break	**0.026**	**0.015**	0.044	0.096	3.99	**0.046**	3.55	0.127	−52	**0.040**	28	**0.009**
Remission vs. First-line	**0.035**	**0.001**	0.064	**0.002**	**8.65**	**<0.001**	**8.65**	**<0.001**	−102	**<0.001**	38	**<0.001**
Remission vs. Second-line	**0.038**	**<0.001**	0.061	**0.006**	6.76	**<0.001**	**7.85**	**<0.001**	−108	**<0.001**	40	**<0.001**
Remission vs. Later-line	**0.042**	**<0.001**	**0.084**	**<0.001**	**9.46**	**<0.001**	**8.43**	**<0.001**	−101	**<0.001**	35	**<0.001**
Remission vs. BSC	**0.132**	**<0.001**	**0.221**	**<0.001**	**21.02**	**<0.001**	**21.27**	**<0.001**	−232	**<0.001**	134	**<0.001**
Neoadjuvant vs. First-line	**0.029**	**0.019**	0.024	0.125	−1.42	0.576	1.51	0.291	−16	0.531	15	0.079
**Non-curative vs. Non-curative**												
Treatment break vs. First-line	0.009	0.576	0.02	0.309	4.66	**0.030**	**5.09**	**0.010**	−51	0.096	10	0.139
First line vs. Second-line	0.003	0.420	−0.002	0.959	−1.89	0.339	−0.79	0.824	−5	0.975	1	0.628
First line vs. Later-line	0.007	0.378	0.02	0.165	**11.56**	0.743	−0.22	0.914	1	0.718	−4	0.638
Later line vs. BSC	**0.090**	**<0.001**	**0.137**	**0.009**	**11.56**	**0.003**	**12.84**	**0.001**	−131	**0.004**	99	**<0.001**

∆ = Difference between means of the two treatment phases (first minus second). Minimal clinically important difference (MID) and statistical differences are bolded: 15D: ≥|0.015|, EQ-5D: ≥|0.08|, EQ-5D Visual Analogue Scale (VAS): ≥|7|, Quality of Life Questionnaire (QLQ)-C30 Global Health Score (GHS) ≥|5|. Abbreviations: BSC = Best Supportive Care.

**Table 4:** The mean health-related quality of life of metastatic colorectal cancer patients compared with that of the standardized general population. Difference from the general population is presented as a negative/positive number that indicates that the patients are on average worse off/better off than the general population.

	Neoadjuvant	Post-Resection	Rehabilitation	Remission	Treatment Break	First-Line	Second-Line	Later-Line	BSC
	*n* = 56	*n* = 58	*n* = 60	*n* = 126	*n* = 87	*n* = 169	*n* = 114	*n* = 105	*n* = 35
**EQ-5D score**	0.030	0.040	0.075 *	0.070 *	0.034	0.018	0.010	−0.013	−0.107 *
**15D score**	**−** **0.023**	**−** 0.035 *	0.002	−0.014	**−** 0.039 *	**−** 0.044 *	−0.052 *	−0.056 *	−0.123 *
Mobility	0.034 *	−0.011	0.016	0.006	−0.058 *	−0.017	−0.028 *	−0.031 *	−0.166 *
Vision	−0.010	−0.045 *	−0.012	−0.001	−0.008	−0.019 *	−0.005	−0.024	−0.065
Hearing	0.021	−0.005	0.002	0.010	0.010	0.015	0.016	0.008	−0.023
Breathing	−0.018	−0.035	−0.032	−0.022	−0.071 *	−0.053 *	−0.084 *	−0.094 *	−0.235 *
Sleeping	−0.022	−0.055	−0.001	−0.036 *	−0.013	−0.036 *	−0.033 *	−0.047 *	−0.047
Eating	−0.015	−0.009	0.004 *	−0.001	−0.006	−0.017 *	0.000	−0.015 *	−0.031
Speech	0.004	*−0.018*	0.003	0.000	−0.011	−0.003	−0.017	−0.009	−0.040
Excretion	−0.045	−0.065 *	−0.008	−0.050 *	−0.047 *	−0.045 *	−0.078 *	−0.074 *	−0.133 *
Usual activities	−0.075	−0.093 *	−0.004	−0.021	−0.100 *	−0.120 *	−0.130 *	−0.119 *	−0.251 *
Mental function	0.013	0.010	0.054 *	0.027 *	0.020	0.021	0.009	0.036 *	0.026
Discomf. and sympt.	0.014	0.004	0.059 *	0.045 *	−0.007	−0.019	−0.025	−0.043 *	−0.118 *
Depression	−0.054 *	−0.029	−0.023	−0.041 *	−0.043 *	−0.079 *	−0.082 *	−0.069 *	−0.105 *
Distress	−0.064 *	−0.049 *	−0.036 *	−0.053 *	−0.052 *	−0.076 *	−0.085 *	−0.079 *	−0.115 *
Vitality	−0.080 *	−0.061 *	−0.013	−0.026 *	−0.078 *	−0.106 *	−0.112 *	−0.129 *	−0.219 *
Sexual activity	−0.163 *	−0.146 *	−0.098 *	−0.148 *	−0.179 *	−0.205 *	−0.204 *	−0.240 *	−0.371 *

* *p*-value < 0.05. Minimal clinically important difference (MID) is marked in bold: 15D: ≥|0.015|, EQ-5D: ≥|0.08|. Abbreviations: BSC = Best Supportive Care, Discomf. and sympt. = Discomfort and symptoms.

## Data Availability

The data collected for this study can be made available to others in a de-identified form after all primary and secondary endpoints have been published, in the presence of a data transfer agreement, and if the purpose of use complies with Finnish legislation. Requests for data sharing can be made to the corresponding author, including a proposal that must be approved by the steering committee.

## Supplementary Materials

The following supporting information can be downloaded at: https://www.mdpi.com/article/10.3390/cancers14071713/s1, Figure S1: Flowchart of individual patients that had metastasectomy/LAT and answered multiple questionnaires (n = 195) during curative treatment phases and/or non-curative treatment phases (i.e., after not re-resectable relapse). Each horizontal line represents an individual with heatmap for 15D index score (if empty, patient did not answer during that treatment phase) and first column denotes mean score of all of the individual’s questionnaires. Statistically significant differences between treatment phases are marked with brackets. Abbreviations: LAT = Local Ablative Therapy, BSC = Best Supportive Care; Figure S2: Flowchart of individual patients that had systemic therapy only and answered multiple questionnaires (n = 95) during curative treatment phases (n = 4) and/or non-curative treatment phases (never-resectable). Each horizontal line represents an individual with heatmap for 15D index score (if empty, patient did not answer during that treatment phase) and first column denotes mean score of all of the individual’s questionnaires. Statistically significant differences between treatment phases are marked with brackets. Abbreviations: LAT = Local Ablative Therapy, BSC = Best Supportive Care; Figure S3: The mean health-related quality of life measured with QLQ-C30 and QLQ-CR29 functioning profile scales during curative intent treatment (A) and during non-curative treatment (B). Abbreviations: BSC = Best Supportive Care; Figure S4: The mean health-related quality of life measured with QLQ-C30 and QLQ-CR29 symptom profile scales before and after metastasectomy (panel A) and in systemic treatments (panel B); Figure S5: Overall survival (OS) from first cross-sectional QoL questionnaire divided by above median (≥0.90) or below median (<0.90) with 15D generic index score, or above median (≥80) or below median (<80) for QLQ-C30 physical function; adjusted hazard ratio (HR) and 95% confidence interval (95% CI) presented. Abbreviations: mCRC = Metastatic Colorectal Cancer; Table S1: Number of patients and questionnaires in the different treatment phases. At most centres, questionnaires were sent out twice a year by mail to all consenting patients still alive; Table S2: Patient demographics for patients included in HRQoL-substudy and patients invited but not responding (did not re-consent and return questionnaires); Table S3: Patient demographics in the different treatment phases; Table S4: Association of demographic factors and index scores during remission phase; Table S5: Association of metastatic sites or metastasectomies and index scores during remission phase.
