# Supplementary material for: Health-Related Quality of Life in Metastatic Colorectal Cancer Patients Treated with Curative Resection and/or Local Ablative Therapy or Systemic Therapy in the Finnish RAXO-Study
Source: Cancers (Basel). 2022 Mar 28;14(7):1713. doi: 10.3390/cancers14071713 (PMC8996978; doi:10.3390/cancers14071713)
Supplement: Supplementary file 1 [file cancers-14-01713-s001.zip › cancers-1560882-supplementary.pdf]

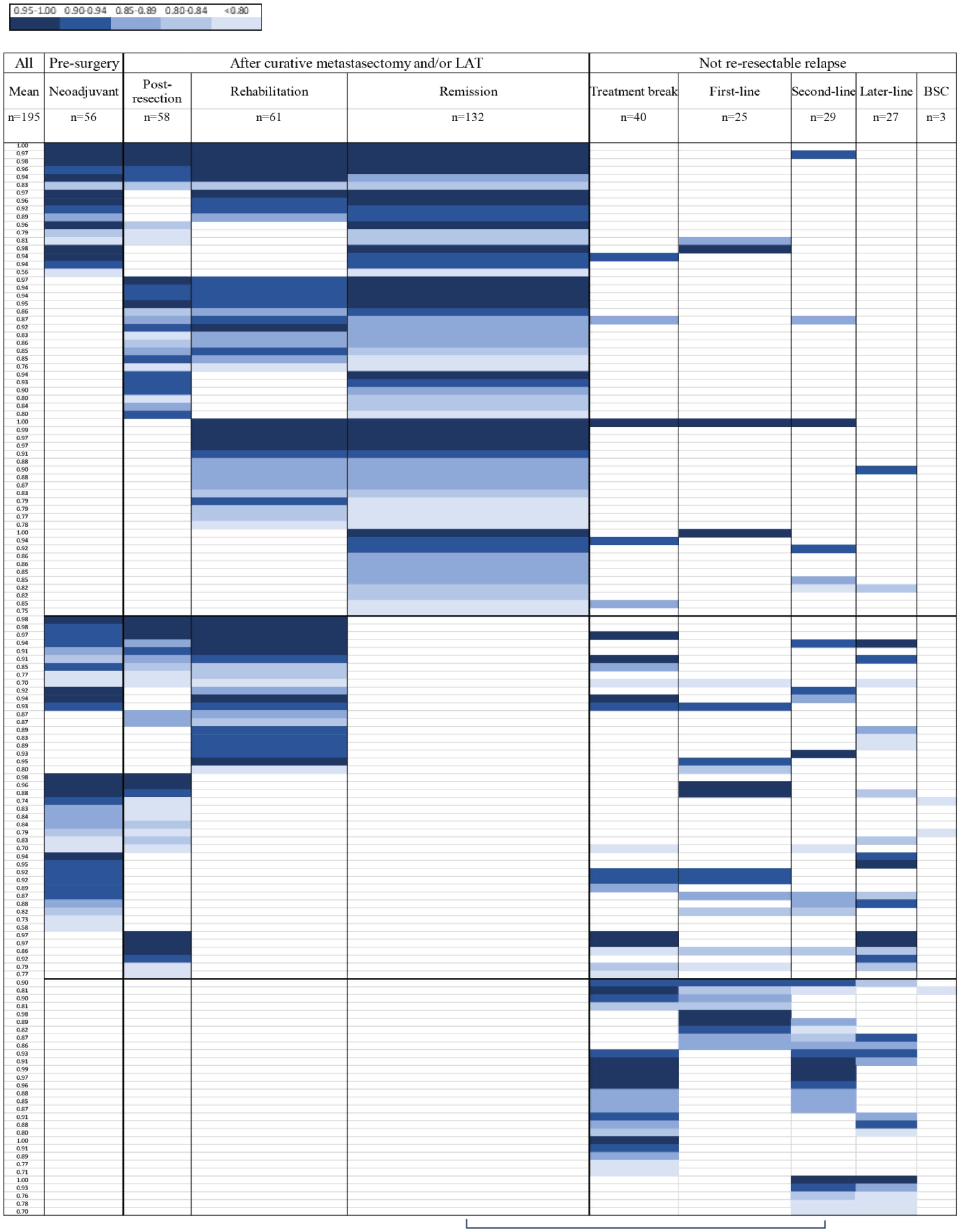

Figure S1. Flowchart of individual patients that had metastasectomy/LAT and answered multiple questionnaires (n=195) during curative treatment phases and/or non-curative treatment phases (i.e., after not re-resectable relapse). Each horizontal line represents an individual with heatmap for 15D index score (if empty patient did not answer during that treatment phase) and first column denotes mean score of the individual's all questionnaires. Statistically significant differences between treatment phases are marked with brackets. Abbreviations: LAT = Local Ablative Therapy, BSC = Best Supportive Care.

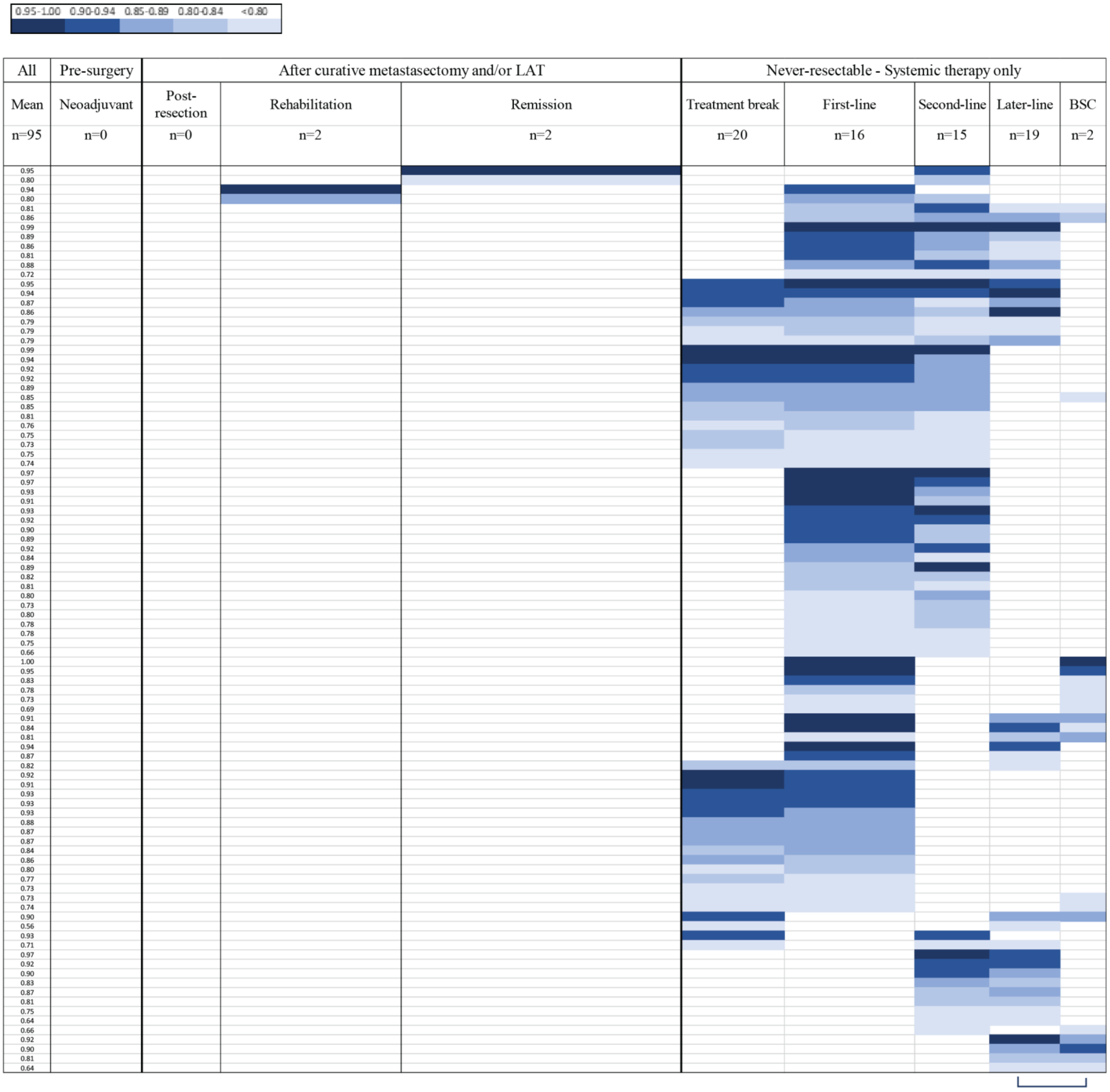

Figure S2. Flowchart of individual patients that had systemic therapy only and answered multiple questionnaires (n=95) during curative treatment phases (n = 4) and/or non-curative treatment phases (never-resectable). Each horizontal line represents an individual with heatmap for 15D index score (if empty patient did not answer during that treatment phase) and first column denotes mean score of the individual's all questionnaires. Statistically significant differences between treatment phases are marked with brackets. Abbreviations: LAT = Local Ablative Therapy, BSC = Best Supportive Care.

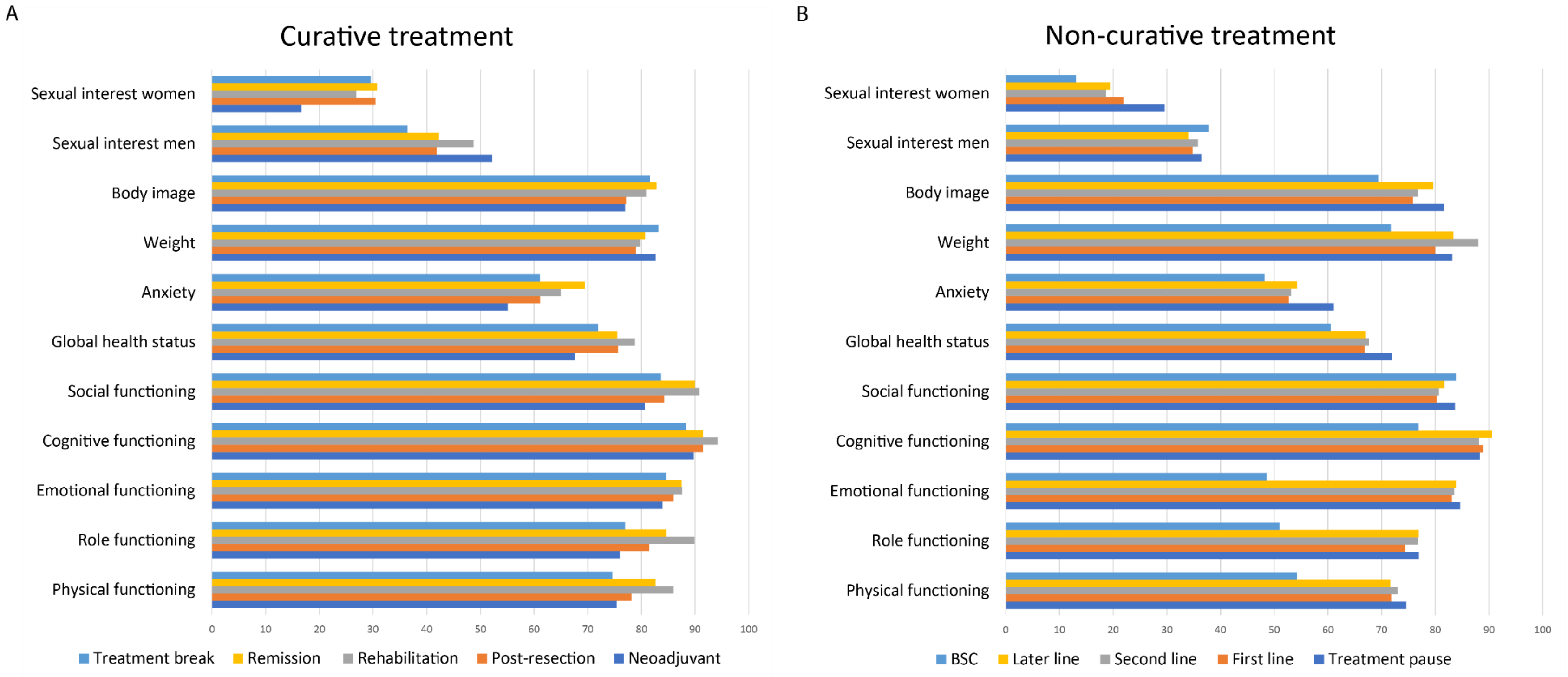

Figure S3. The mean health-related quality of life measured with QLQ-C30 and QLQ-CR29 functioning profile scales during curative intent treatment (A) and during non-curative treatment (B). Abbreviations: BSC = Best Supportive Care.

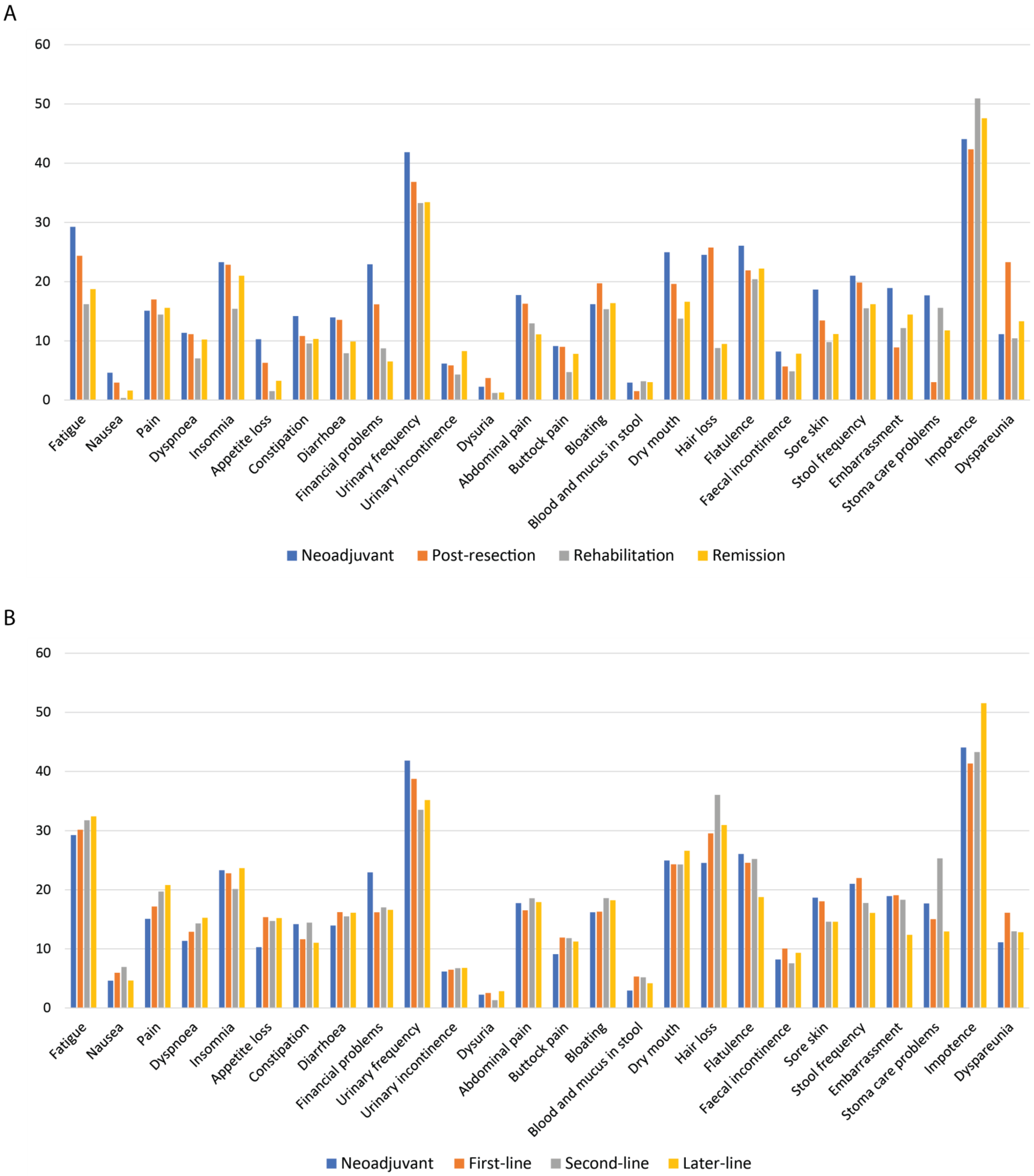

Figure S4. The mean health-related quality of life measured with QLQ-C30 and QLQ-CR29 symptom profile scales before and after metastasectomy (panel A) and in systemic treatments (panel B).

A

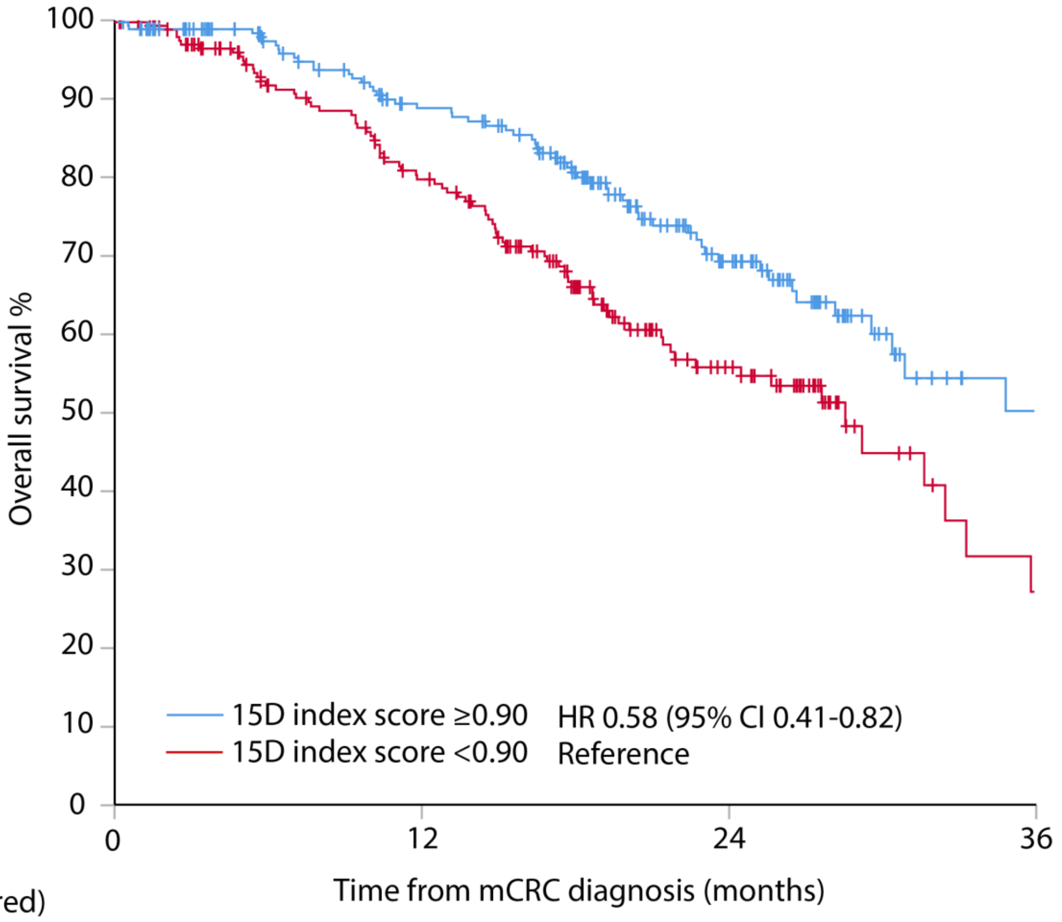

Number at risk (censored)

15D index score ≥0.90	223 (44)	158 (105)	69 (153)	12 (160)
15D index score <0.90	219 (38)	143 (95)	51 (131)	6 (135)

B

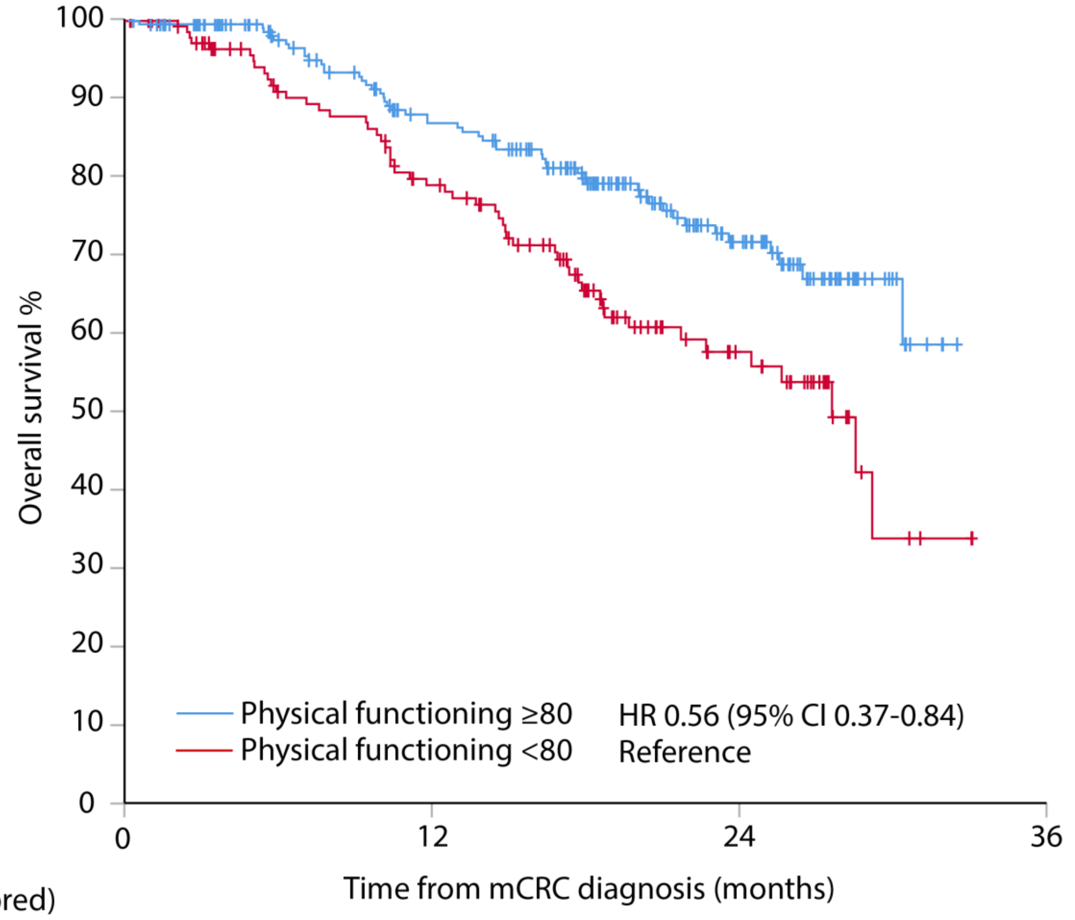

Number at risk (censored)

Physical functioning ≥0.80	234 (53)	156 (126)	62 (184)	0 (184)
Physical functioning <0.80	149 (26)	96 (70)	31 (96)	0 (96)

Figure S5. Overall survival (OS) from first cross-sectional QoL questionnaire divided by above median (≥0.90) or below median (<0.90) with 15D generic index score or above median (≥80) or below median (<80) for QLQ-C30 physical function; adjusted hazard ratio (HR) and 95% confidence interval (95% CI) presented. Abbreviations: mCRC = Metastatic Colorectal Cancer.

Table S1. Number of patients and questionnaires in the different treatment phases. At most centres, questionnaires were sent out twice a year by mail to all consenting patients still alive

<table>
<tr><td rowspan="2"></td><td rowspan="2">Treatment</td><td colspan="5">Patients</td><td>Questionnaires</td></tr>
<tr><td>All
n=444*</td><td colspan="2">Metastasectomy
n=247*</td><td colspan="2">Systemic therapy
n=197*</td><td>n=1751∫</td></tr>
<tr><td rowspan="4">Curative ¥
n=138+62+4</td><td>Neoadjuvant</td><td>54</td><td>54</td><td rowspan="4">138+62 ¥</td><td>-</td><td rowspan="4">0+4 ¥</td><td>77</td></tr>
<tr><td>Post-resection</td><td>58</td><td>58</td><td>-</td><td>82</td></tr>
<tr><td>Rehabilitation</td><td>61</td><td>59</td><td>2</td><td>109</td></tr>
<tr><td>Remission</td><td>132</td><td>130</td><td>2</td><td>366</td></tr>
<tr><td></td><td></td><td></td><td colspan="2">Not re-resectable relapse n=109</td><td colspan="2">Long-term remission with systemic therapy [illegible]</td><td></td></tr>
<tr><td rowspan="5">Non-curative ₵
n=62+47+4+193</td><td>Treatment</td><td>87</td><td>40</td><td rowspan="5">62+47 ₵</td><td>47</td><td rowspan="5">4+193 ₵</td><td>200</td></tr>
<tr><td>First-line</td><td>169</td><td>28</td><td>141</td><td>470</td></tr>
<tr><td>Second-line</td><td>115</td><td>40</td><td>75</td><td>209</td></tr>
<tr><td>Later-line</td><td>106</td><td>54</td><td>52</td><td>194</td></tr>
<tr><td>BSC</td><td>35</td><td>10</td><td>25</td><td>44</td></tr>
</table>

* 188 (42%) patients answered one questionnaire and 256 (58%) patients answered multiple (2-13) questionnaires during the treatment phases. If multiple questionnaires were answered during one phase the mean value for that patient was used.
∫ Some patients have multiple answers during one treatment phase
¥ Curative: patients rendered tumour free with systemic therapy n=2 or metastasectomy/LAT n= 200 (of which 138 have answered questionnaires only in curative phase and 62 in curative phase and then later in noncurative phase, as not re-resectable at relapse)
₵ Non-curative: relapse after metastasectomy/LAT that was not re-resectable and treated with systemic therapy n=62+47, or systemic therapy only n=193+4
Abbreviations: LAT = Local Ablative Therapy, BSC = Best Supportive Care.

Table S2. Patient demographics for patients included in HRQoL-substudy and patients invited but not responding (did not return questionnaires).

		Metastasectomy/LAT				Systemic therapy only			
		Included in HRQoL-substudy		Invited but not responding		Included in HRQoL-substudy		Invited but not responding	
		247	96 %	9	4 %	197	89 %	25	11 %
Age	Median (range)	64.3	(25.0-81.5)	76.6	(57.6-77.9)	66.2	(24.3-87.9)	67.2	(39.1-84.9)
	≤70	189	77 %*	2	22 %	133	68 %	17	68 %
	>70	58	24 %	7	78 %*	64	33 %	8	32 %
Sex	Male	142	58 %	7	78 %	116	59 %	17	68 %
	Female	105	43 %	2	22 %	81	41 %	8	32 %
ECOG	PS 0	115	47 %*	1	11 %	57	29 %*	2	8 %
	PS 1	120	49 %	6	67 %	115	58 %	15	60 %
	PS 2-3	12	5 %	2	22 %*	25	13 %	8	32 %*
Charlson comorbidity index	0	204	83 %	8	89 %	156	79 %	17	68 %
	1 to 2	42	17 %	1	11 %	40	20 %	7	28 %
	3 to 5	1	0 %	0	0 %	1	1 %	1	4 %
Smoking status	Never or former smoker	159	87 %	6	86 %	125	83 %	19	91 %
	Current smoker	23	13 %	1	14 %	25	17 %	2	10 %
BMI	≥20	227	92 %*	6	67 %	185	94 %	23	92 %
	<20	20	8 %	3	33 %*	12	6 %	2	8 %
Presentation of metastases	Synchronous	155	63 %	5	56 %	143	73 %	19	76 %
	Early metachronous§	21	9 %	1	11 %	17	9 %	1	4 %
	Late metachronous	71	29 %	3	33 %	37	19 %	5	20 %
Primary location	Right colon	50	20 %	2	22 %	59	30 %	10	40 %
	Left colon	116	47 %	2	22 %	65	33 %	4	16 %
	Rectum	81	33 %	5	56 %	72	37 %	10	40 %
	Multiple	0	0 %	0	0 %	1	1 %	1	4 %
Surgery primary tumour	Operated upfront	196	79 %	6	67 %	118	60 %*	9	36 %
	Later or never operated	51	21 %	3	33 %	79	40 %	16	64 %*
Adjuvant therapy	No adjuvant	177	72 %	6	67 %	161	82 %	21	84 %
	Fluoropyrimidine	27	11 %	1	11 %	18	9 %	2	8 %
	Oxaliplatin-based	43	17 %	2	22 %	18	9 %	2	8 %
Radiotherapy for primary	No	208	84 %	7	78 %	172	87 %	21	84 %
	Preop 5x5Gy	22	9 %	1	11 %	5	3 %	1	4 %
	Chemoradiation	13	5 %	1	11 %	17	9 %	1	4 %
	Palliative	4	2 %	0	0 %	3	2 %	2	8 %*
Metastatic sites	1 site	195	79 %	6	67 %	89	45 %	10	40 %
	2 sites	43	17 %	2	22 %	70	36 %	8	32 %
	3 to 6 sites	9	4 %	1	11 %	38	19 %	7	28 %
Liver metastases	No liver metastases	46	19 %	2	22 %	72	37 %	5	20 %
	Liver metastases	201	81 %	7	78 %	125	64 %	20	80 %
Lung metastases	No lung metastases	211	85 %	8	89 %	114	58 %	15	60 %
	Lung metastases	36	15 %	1	11 %	83	42 %	10	40 %
Peritoneal metastases	No peritoneal metastases	226	92 %	8	89 %	160	81 %	20	80 %
	Peritoneal metastases	21	9 %	1	11 %	37	19 %	5	20 %
Distant lymph node metastases	No distant lymph node metastases	230	93 %	9	100 %	119	60 %	16	64 %
	Distant lymph node metastases	17	7 %	0	0 %	78	40 %	9	36 %
Other metastases	No other metastases	222	90 %*	6	67 %	169	86 %	22	88 %
	Other metastases	25	10 %	3	33 %*	28	14 %	3	12 %
Molecular status	*RAS*/*BRAF* wildtype	94	43 %	4	50 %	59	32 %	5	21 %
	KRAS/*NRAS* mutant	120	54 %	3	38 %	102	56 %	13	54 %
	BRAF mutant	7	3 %	1	13 %	22	12 %	6	25 %

§ early metachronous = 2-12 months from diagnosis of metastatic disease, late metachronous = more than 12 months from diagnosis. Abbreviations: LAT = Local Ablative Therapy, HRQoL = Health-Related Quality of Life, ECOG PS = Eastern Cooperative Oncology Group Performance Status, BMI = Body Mass Index. *KRAS* = Kirsten rat sarcoma viral oncogene homolog, *NRAS* = Neuroblastoma rat sarcoma viral oncogene homolog, *BRAF* = v-raf murine sarcoma viral oncogene homolog B1. Statistical differences denoted with *

Table S3. Patient demographics in the different treatment phases

		Neoadjuvant n= 54		Post-resection n=59		Rehabilitation n=62		Remission n= 112		Treatment break n= 87		First-line n=169		Second-line n=116		Later-line n= 106		BSC n=35	
Age	Median (range)	62.9	(25-82)	64.3	(42-77)	66	(42-80)	64.7	(33-80)	64.6	(44-88)	65.9	(24-88)	63.9	(24-85)	64.2	(25-81)	73	(38-84)
	≤70	43	80 %	44	75 %	45	73 %	99	75 %	64	74 %	121	72 %	87	75 %	79	75 %	13	37 %
	>70	11	20 %	15	25 %	17	27 %	33	25 %	23	26 %	48	28 %	29	25 %	27	26 %	22	63 %
Sex	Male	34	63 %	35	59 %	39	63 %	78	59 %	58	67 %	96	57 %	65	56 %	63	59 %	16	46 %
	Female	20	37 %	24	41 %	23	37 %	54	41 %	29	33 %	73	43 %	51	44 %	43	41 %	19	54 %
ECOG	PS 0	22	41 %	24	41 %	35	57 %	65	49 %	38	44 %	50	30 %	44	38 %	46	43 %	5	14 %
	PS1	31	57 %	31	53 %	27	44 %	62	47 %	44	51 %	97	57 %	64	55 %	53	50 %	26	74 %
	PS 2-3	1	2 %	4	7 %	0	0 %	5	4 %	5	6 %	22	13 %	8	7 %	7	7 %	4	11 %
Charlson comorbidity index	0	45	83 %	48	81 %	54	87 %	112	85 %	71	82 %	140	83 %	96	83 %	85	80 %	21	60 %
	1 to 2	9	17 %	10	17 %	8	13 %	20	15 %	15	17 %	28	17 %	20	17 %	21	20 %	13	37 %
	3 to 5	0	0 %	1	2 %	0	0 %	0	0 %	1	1 %	1	1 %	0	0 %	0	0 %	1	3 %
Smoking	No	31	89 %	37	95 %	48	94 %	84	92 %	62	86 %	111	84 %	80	92 %	71	83 %	22	92 %
	Yes	4	11 %	2	5 %	3	6 %	7	8 %	10	14 %	22	17 %	7	8 %	15	17 %	2	8 %
BMI	≥20	50	93 %	56	95 %	60	97 %	123	93 %	82	94 %	153	91 %	108	93 %	100	94 %	33	94 %
	<20	4	7 %	3	5 %	2	3 %	9	7 %	5	6 %	16	10 %	8	7 %	6	6 %	2	6 %
Presentation§	Synchronous	37	69 %	40	68 %	36	58 %	78	59 %	59	68 %	128	76 %	85	73 %	74	70 %	22	63 %
	Early metachronous	6	11 %	4	7 %	5	8 %	12	9 %	4	5 %	12	7 %	8	7 %	13	12 %	3	9 %
	Late metachronous	11	20 %	15	25 %	21	34 %	42	32 %	24	28 %	29	17 %	23	20 %	19	18 %	10	29 %
Primary tumour	Right colon	11	20 %	11	19 %	17	27 %	25	19 %	21	24 %	52	31 %	32	28 %	19	18 %	7	20 %
	Left colon	31	57 %	28	48 %	30	48 %	65	49 %	31	36 %	59	35 %	44	38 %	47	44 %	15	43 %
	Rectum	12	22 %	20	34 %	15	24 %	42	32 %	35	40 %	58	34 %	40	35 %	40	38 %	12	34 %
	Multiple	0	0 %	0	0 %	0	0 %	0	0 %	0	0 %	0	0 %	0	0 %	0	0 %	1	3 %
Surgery primary tumour	Operated upfront	45	83 %	44	75 %	51	82 %	113	86 %	60	69 %	100	59 %	74	64 %	69	65 %	25	71 %
	Not operated or later	9	17 %	15	25 %	11	18 %	19	14 %	27	31 %	69	41 %	42	36 %	37	35 %	10	29 %
Prior adjuvant therapy	No adjuvant	40	74 %	43	73 %	43	69 %	92	70 %	64	74 %	140	83 %	95	82 %	86	81 %	23	66 %
	Fluoropyrimidine	6	11 %	9	15 %	8	13 %	14	11 %	7	8 %	10	6 %	13	11 %	12	11 %	7	20 %
	Oxaliplatin-based	8	15 %	7	12 %	11	18 %	26	20 %	16	18 %	19	11 %	8	7 %	8	8 %	5	14 %
Radiotherapy for rectal	No	49	91 %	48	81 %	53	86 %	111	84 %	77	89 %	150	89 %	102	88 %	89	84 %	26	74 %
	Preop 5x5Gy	3	6 %	5	9 %	6	10 %	13	10 %	4	5 %	5	3 %	6	5 %	7	7 %	4	11 %
	Chemoradiation	1	2 %	4	7 %	2	3 %	6	5 %	4	5 %	12	7 %	7	6 %	10	9 %	5	14 %
	Palliative	1	2 %	2	3 %	1	2 %	2	2 %	2	2 %	2	1 %	1	1 %	0	0 %	0	0 %
Metastatic sites	1	39	72 %	45	76 %	53	86 %	114	86 %	49	56 %	75	44 %	61	53 %	67	63 %	18	51 %
	2	12	22 %	10	17 %	7	11 %	16	12 %	30	35 %	60	36 %	37	32 %	28	26 %	10	29 %
	3 to 6	3	6 %	4	7 %	2	3 %	2	2 %	8	9 %	34	20 %	18	16 %	11	10 %	7	20 %
	Liver	47	87 %	48	81 %	49	79 %	108	82 %	63	72 %	116	69 %	78	67 %	79	75 %	23	66 %
	Lung	7	13 %	9	15 %	5	8 %	14	11 %	26	30 %	68	40 %	37	32 %	34	32 %	17	49 %
	Peritoneal	4	7 %	6	10 %	8	13 %	11	8 %	8	9 %	34	20 %	24	21 %	12	11 %	4	11 %
	Distant lymph nodes	6	11 %	5	9 %	6	10 %	8	6 %	26	30 %	60	36 %	38	33 %	21	20 %	8	23 %
	Other	5	9 %	6	10 %	4	7 %	8	6 %	8	9 %	26	15 %	15	13 %	10	9 %	6	17 %
Molecular status	*RAS*/*BRAF* wt	27	50 %	29	50 %	28	46 %	52	48 %	32	37 %	54	34 %	33	30 %	32	32 %	14	42 %
	KRAS/*NRAS* mt	24	44 %	27	47 %	29	48 %	53	49 %	49	57 %	85	53 %	67	61 %	62	63 %	17	52 %
	BRAF mt	3	6 %	2	3 %	4	7 %	3	3 %	5	6 %	22	14 %	10	9 %	5	5 %	2	6 %
	BRAF wt tested	40	93 %	47	96 %	45	92 %	76	96 %	67	93 %	115	84 %	81	89 %	62	93 %	21	91 %
	BRAF mt tested	3	7 %	2	4 %	4	8 %	3	4 %	5	7 %	22	16 %	10	11 %	5	8 %	2	9 %

§ early metachronous = 2-12 months from diagnosis of metastatic disease, late metachronous = more than 12 months from diagnosis. Abbreviations: BSC = Best Supportive Care, ECOG PS = Eastern Cooperative Oncology Group Performance Status, BMI = Body Mass Index. *KRAS* = Kirsten rat sarcoma viral oncogene homolog, *NRAS* = Neuroblastoma rat sarcoma viral oncogene homolog, *BRAF* = v-raf murine sarcoma viral oncogene homolog B1.

Table S4. Association of demographic factors and index scores during remission phase

		n	15D score		P-value	EQ-5D score		P-value	VAS score		P-value	QLQ-C30 GHS		P-value
			Mean	SD		Mean	SD		Mean	SD		Mean	SD	
Age	≤70	99	0.897	0.094		0.881	0.142		79.1	14.8		77.5	16.8	
	>70	33	0.888	0.087		0.849	0.127		74.1	15.2		69.3	23.3	*
Sex	Male	78	0.889	0.091		0.874	0.143		77.2	15.2		74.2	20.3	
	Female	54	0.903	0.095		0.872	0.133		78.7	14.8		77.3	16.5	
ECOG	PS 0	65	0.924	0.069		0.911	0.132		79.8	13.8		77.7	19.5	
	PS 1	62	0.875	0.089	*	0.842	0.137		76.4	16.1		73.6	18.3	
	PS 2-3	5	0.759	0.194	*	0.794	0.138	*	70.4	10.9		71.9	15.0	
Charlson index	0	112	0.901	0.093		0.883	0.134		79.0	14.6		77.8	17.0	
	1 to 5	20	0.861	0.083		0.818	0.154		72.0	15.8		62.8	23.3	*
Number of comorbidities	No	43	0.938	0.054		0.924	0.122		83.5	11.4		81.6	14.4	
	1 to 3	80	0.881	0.098	*	0.853	0.135	*	75.5	16.0	*	73.3	19.7	*
	4 to 12	9	0.814	0.094	*	0.804	0.180	*	71.2	13.6	*	65.2	22.6	*
Number of regular	0-3	90	0.911	0.077		0.880	0.137		79.7	13.7		77.9	17.9	
drugs	4-12	42	0.859	0.112	*	0.860	0.143		73.9	16.9	*	70.4	19.8	*
BMI	<20	9	0.901	0.169		0.934	0.123		86.2	10.7		87.1	11.1	
	20-30	99	0.907	0.081		0.889	0.133	*	79.3	14.1	*	77.6	17.6	*
	>30	24	0.844	0.083	*	0.782	0.133	*	68.9	16.4	*	62.4	20.1	*
Smoking	Never	56	0.894	0.084		0.863	0.141		77.0	14.4		73.9	20.9	
	Ex-smoker	28	0.879	0.124		0.868	0.137		78.3	16.5		73.4	17.5	
	Smoker	7	0.929	0.047		0.953	0.056		79.8	8.9		78.5	9.6	
Presentation	Synchronous	78	0.901	0.088		0.882	0.132		79.3	14.5		77.4	16.6	
	Metachronous	54	0.886	0.098		0.862	0.149		75.8	15.5		72.7	21.5	
Primary tumour	Right colon	25	0.903	0.088		0.852	0.142		74.0	16.9		74.7	17.7	
	Left colon	65	0.901	0.079		0.878	0.140		79.1	15.0		75.3	20.4	
	Rectum	42	0.882	0.112		0.878	0.136		78.1	13.7		76.2	17.3	
TNM stage	I	11	0.879	0.079		0.879	0.123		77.0	13.6		70.0	26.4	
	II	22	0.903	0.076		0.864	0.156		75.7	17.5		74.9	19.7	
	III	33	0.889	0.107		0.871	0.144		76.2	14.2		74.3	16.5	
	IV	66	0.898	0.092		0.877	0.135		79.6	14.8		77.3	18.4	
Molecular status	*RAS* +/- *BRAF* wildtype	65	0.881	0.105		0.864	0.141		75.9	16.2		71.8	20.4	
	RAS mutation	53	0.900	0.079		0.871	0.142		79.5	12.3	*	77.1	16.8	
	BRAF mutation	3	0.926	0.076		0.879	0.209		61.6	27.5		80.6	19.2	
Mismatch repair status	proficient MMR	39	0.874	0.117		0.884	0.153		76.4	15.7		73.0	22.9	
	deficient MMR	3	0.880	0.043		0.791	0.143		63.4	25.9		66.2	17.4	
	Not tested	90	0.905	0.080		0.872	0.132		79.0	14.1		76.9	16.8	
Primary surgery	Abdominoperineal resection	7	0.877	0.122		0.869	0.130		77.1	22.2		72.4	26.6	
	Anterior resection	21	0.890	0.090		0.872	0.142		79.9	12.9		77.9	17.6	
	Anterior resection (stoma)	16	0.883	0.138		0.896	0.134		78.9	11.6		76.9	13.7	
	Bowel resection	2	0.901	0.098		0.898	0.144		77.3	31.5		80.2	19.2	
	Colectomy	3	0.848	0.058		0.598	0.112	*	55.7	12.8	*	50.4	15.4	*
	Haartman's operation (stoma)	1	0.747	.		0.703	.		50.0	.		58.3	.	
	Left hemicolectomy	15	0.889	0.079		0.906	0.134		76.7	16.5		70.6	26.6	
	Right hemicolectomy	21	0.903	0.091		0.847	0.148		73.5	16.8		74.4	18.9	
	Sigmaresection	41	0.906	0.079		0.890	0.126		80.7	12.6		77.8	17.1	
	Subtotal colectomy	4	0.927	0.060		0.880	0.120		84.3	11.0		83.3	7.2	
Surgery primary tumour	Operated upfront	113	0.900	0.091		0.878	0.137		78.6	14.8		76.2	18.7	
	Not operated or later	19	0.863	0.095		0.846	0.151		73.9	15.6		71.2	19.3	
Adjuvant therapy	Not given	92	0.903	0.083		0.876	0.140		79.2	14.7		75.7	19.8	
	Fluoropyridine +/- oxaliplatin	40	0.883	0.105		0.869	0.137		75.8	15.3		75.1	17.4	
Radiotherapy	Preoperative 5x5Gy	13	0.833	0.139	*	0.838	0.124	*	70.3	9.9	*	72.0	13.4	*
	Chemoradiation	6	0.949	0.064		0.954	0.112		88.8	5.1		86.3	15.8	
	Palliative	2	0.694	0.066	*	0.682	0.013		47.5	24.7	*	35.4	8.8	*
	No	111	0.903	0.080		0.877	0.139		78.7	14.8	*	76.0	18.7	*

Abbreviations: VAS = Visual Analogue Scale, GHS = Global Health Status, ECOG PS = Eastern Cooperative Oncology Group Performance Status, BMI = Body Mass Index.

Table S5. Association of metastatic sites or metastasectomies and index scores during remission phase

		n=	15D score		P-	EQ-5D score		P-	VAS		P-	GHS		P-
		132	Mean	SD	value	Mean	SD	value	Mean	SD	value	Mean	SD	value
Metastatic sites	1 site at baseline	114	0.897	0.092		0.874	0.138		78.5	14.1		75.9	18.0	
	2 sites at baseline	16	0.880	0.096		0.865	0.151		71.7	19.6		70.8	25.0	
	3 to 6 sites at baseline	2	0.899	0.101		0.924	0.108		89.5	14.1		88.5	7.4	
	1 site during trajectory	90	0.896	0.096		0.874	0.136		78.2	14.7		75.6	17.4	
	2 sites during trajectory	1	0.898	0.079		0.888	0.142		76.9	15.1		75.5	22.7	
	3 to 9 sites during trajectory	11	0.878	0.104		0.829	0.152		77.4	18.0		74.7	20.8	
Liver metastases	Absent at baseline	24	0.900	0.112		0.911	0.115		79.1	16.7		84.5	11.7	*
	Present at baseline	108	0.894	0.088		0.866	0.142		77.6	14.6		73.6	19.5	
	Absent during trajectory	21	0.895	0.118		0.911	0.114		79.3	17.2		83.9	12.2	*
	Present during trajectory	111	0.895	0.087		0.867	0.142		77.6	14.6		74.0	19.4	
Lung metastases	Absent at baseline	118	0.897	0.084		0.866	0.140		77.6	14.8		75.2	17.8	
	Present at baseline	14	0.880	0.149		0.942	0.108		79.9	17.2		78.3	27.2	
	Absent during trajectory	100	0.896	0.084		0.867	0.138		77.4	15.1		75.0	17.3	
	Present during trajectory	32	0.891	0.115		0.898	0.141		79.4	14.8		76.9	23.6	
Peritoneal metastases	Absent at baseline	121	0.893	0.094		0.871	0.140		78.1	14.6		74.9	19.3	
	Present at baseline	11	0.912	0.064		0.904	0.127		74.6	19.8		82.5	9.8	
	Absent during trajectory	120	0.893	0.095		0.872	0.140		78.2	14.6		74.9	19.4	
	Present during trajectory	12	0.911	0.061		0.895	0.125		74.3	18.8		82.1	9.4	
Distant lymph nodes	Absent at baseline	124	0.893	0.094		0.873	0.140		77.5	15.1		75.0	19.0	
	Present at baseline	8	0.932	0.055		0.883	0.120		84.2	11.5		84.1	12.5	
	Absent during trajectory	120	0.893	0.092		0.876	0.138		77.5	15.0		75.1	18.9	
	Present during trajectory	12	0.914	0.092		0.848	0.144		81.1	14.9		79.5	18.6	
Other metastatic sites	Absent at baseline	124	0.898	0.092		0.877	0.137		78.5	14.3		75.6	18.9	
	Present at baseline	8	0.847	0.085		0.828	0.161		68.1	21.6		73.8	18.2	
	Absent during trajectory	108	0.894	0.095		0.875	0.139		77.8	14.6		74.7	19.2	
	Present during trajectory	24	0.899	0.082		0.867	0.139		78.3	16.8		79.0	17.0	
Metastasectomies	1	87	0.894	0.099		0.861	0.147		77.7	15.8		75.0	18.3	
	2	26	0.893	0.088		0.883	0.130		79.8	14.4		73.8	23.8	
	3	13	0.909	0.072		0.912	0.116		78.5	10.1		81.4	14.6	
	4	4	0.872	0.014		0.931	0.063		66.1	12.2		77.8	10.4	
Liver procedure	Not done	23	0.898	0.113		0.912	0.111		80.1	16.5		84.5	11.9	
	Performed	109	0.894	0.088		0.866	0.143		77.4	14.7		73.7	19.5	*
	1 procedure	94	0.893	0.091		0.862	0.148		77.3	15.1		72.9	20.3	
	2 procedures	12	0.904	0.067		0.885	0.117		79.2	12.3		77.9	12.9	
	3 procedures	2	0.887	0.089		0.919	0.115		82.4	10.8		87.2	14.2	
	5 procedures	1	0.869	.		0.850	.		58.0	.		66.7	.	
Lung procedure	Not done	107	0.897	0.084		0.861	0.139		77.6	15.0		75.1	17.4	
	Performed	25	0.887	0.124		0.928	0.123	*	78.8	15.1		77.3	24.5	
	1 procedure	18	0.874	0.141		0.913	0.139		76.9	17.0		73.9	27.9	
	2 procedures	6	0.927	0.063		0.970	0.074		84.7	8.0		84.3	12.0	
	3 procedures	1	0.883	.		0.925	.		75.5	.		91.7	.	
Cytoreductive surgery	Not done	120	0.893	0.095		0.872	0.140		78.2	14.6		74.9	19.4	
	Performed	12	0.911	0.061		0.895	0.125		74.3	18.8		82.1	9.4	
	1 procedure	7	0.902	0.069		0.881	0.137		66.5	20.6		80.9	12.0	
	2 procedures	4	0.940	0.041		0.949	0.102		86.5	12.3		84.5	6.7	
	3 procedures	1	0.853	.		0.761	.		73.0	.		79.2	.	
Other resections	Not done	118	0.896	0.092		0.877	0.137		78.5	14.5		75.5	18.7	
	Performed	14	0.885	0.096		0.844	0.154		71.9	18.8		75.6	21.2	
	1 procedure	10	0.885	0.097		0.843	0.164		70.4	21.1		77.9	21.3	
	2 procedures	4	0.884	0.108		0.848	0.155		76.3	11.5		68.8	24.0	

Abbreviations: VAS = Visual Analogue Scale, GHS = Global Health Status.
